# Semiconductor‐Bimetallic Plasmonic Heterojunction ZnO−Ag−Cu as Reusable SERS Substrate with Attomolar Detection Limit

**DOI:** 10.1002/asia.202401580

**Published:** 2025-02-18

**Authors:** Rojalin Behera, Amit Nag

**Affiliations:** ^1^ Department of Chemistry Birla Institute of Technology and Science (BITS) Pilani Hyderabad Campus Jawahar Nagar, Kapra Mandal, Hyderabad 500078 India

**Keywords:** SERS, ZnO−Ag−Cu, Attomolar, Rhodamine 6G, Reusability

## Abstract

Semiconductor‐bimetallic ZnO−Ag−Cu (ZAC) heterojunction of different compositions were fabricated as highly sensitive SERS substrates. ZnO nanorods were synthesized using a facile hydrothermal route. ZAC composites were synthesized via impregnation method by keeping ZnO content same and varying the mole fractions of Ag and Cu. The ZnO matrix, known for its stability and photocatalytic properties, was decorated with Ag and Cu nanoparticles to enhance plasmonic activity and boost SERS. Introducing semiconductor oxide as SERS substrate reduces the substrate cost due to its self‐cleaning property upon exposure to UV light. When SERS activity of ZAC composites were compared with either ZnO−Ag (ZA) or ZnO−Cu (ZC) composites, the best SERS performance was recorded with ZAC55, where the Ag and Cu content are same. ZAC55 produced a SERS enhancement factor of 6.2×10⁶ and a limit of detection of 10^−18^ M and 10^−15^ M for the analyte molecules Rhodamine 6 G (R6G) and Methylene Blue (MB), respectively, using 532 nm laser excitation. The enhanced SERS performance is attributed to the synergistic effects of ZnO, Ag, and Cu, unveiling ZAC55 as a promising next‐generation SERS substrate. Along with remarkable sensitivity, ZAC55 showed promising reusability and reproducibility, indicating its potential for practical applications in chemical sensing.

## Graphical abstract

Easily synthesized, economic, UV‐cleanable semiconductor‐bimetallic plasmonic ZnO‐Ag‐Cu (ZAC) composite shows extraordinary SERS efficiency by detecting analyte molecule R6G distinctly, at attomolar level. The synergy between the metal additives and ZnO results in enhanced plasmonic efficiency, improved charge transfer and useful photocatalytic property, rendering ZnO‐Ag‐Cu as a highly sensitive and self‐cleanable SERS substrate.

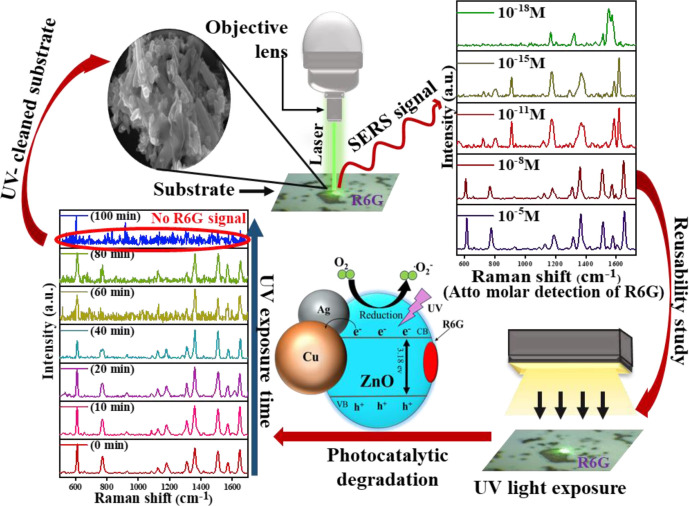

## 1 Introduction

With the benefits of high sensitivity, fingerprint specificity, non‐destructive detection, rapid detection speed, and the ability to integrate and miniaturize the entire system, SERS is a powerful spectroscopic technology that accomplishes ultra‐trace detection by magnifying the Raman signal of molecules adsorbed onto the nanostructured surfaces.^[1]^ It is an economical, portable sensor technology with an abundance of numerous applications, including chemical and biological sensing, medical diagnosis, environmental analysis, food safety, and artwork restoration.^[2]^ The two primary mechanisms of SERS are commonly understood to be electromagnetic (EM) and chemical (CM) enhancement. EM enhancement refers to the amplification of the Raman signal through the intense electromagnetic field produced by localized surface plasmon resonance (LSPR) in metallic nanoscale structures. In contrast, CM enhancement, which is more intricate, involves charge transfer interactions between the SERS substrate and the adsorbed molecules, leading to increased Raman signal intensity.[^3^, ^4^]

Noble metal nanoparticles made of gold (Au) and silver (Ag) are widely employed as efficient SERS substrates due to their ability to produce strong electromagnetic fields through LSPR. This enables highly sensitive detection, even at the single‐molecule level, with significant SERS enhancement factors (EF) in the range of 10^6^ to 10^7^.^[5]^ Despite their effectiveness, noble metal SERS substrates have several drawbacks, including limited stability, susceptibility to oxidation mainly with Ag, high cost of Au and poor reproducibility. These challenges drive the search for alternative hybrid materials to be used as effective SERS substrates, but with lesser oxidation of Ag and more economic, but maintaining high stability and reproducibility. In recent years, semiconductor‐based SERS substrates have garnered significant attention due to their chemical stability, wide range of available materials, and lower cost compared to noble metals.[^6^, ^7^] Also, semiconductors possess excellent photocatalytic properties, making the substrates self‐cleanable under exposure of light and improving the reproducibility of SERS signals.[^8^, ^9^] Additionally, they offer capabilities in sensing and optoelectronic applications, along with superior biocompatibility, making them promising candidates for biological applications. However, the lower SERS enhancement efficiency of semiconductor‐based SERS substrates (typically 10–10^2^) remains a major challenge in the field.^[10]^ To address this, integrating semiconductors with noble metals has been proposed as an effective strategy, leveraging the synergistic effects of LSPR and charge transfer (CT) to significantly improve Raman signal detection.^[11]^

Semiconductor oxide‐metal heterojunction SERS substrates provide several advantages due to the synergy between the metal and the semiconductor oxide.[^12^, ^13^] The metal nanoparticles generate strong localized electric fields, amplifying the Raman signals of nearby molecules, while the semiconductor oxide offers additional charge‐transfer routes, leading to chemical enhancement. Moreover, semiconductor oxides can broaden the light absorption range, making them more responsive to different excitation wavelengths, and their photocatalytic properties allow for self‐cleaning surfaces, which is beneficial for sensor reusability. Various semiconductors, such as Fe₃O₄, Cu₂O, MnO₂, TiO₂, and ZnO, have been successfully combined with noble metal nanomaterials, achieving significant Raman enhancement through the synergistic effects of EM and CT mechanisms. Among these, the photocatalytic properties of TiO₂ and ZnO enable the self‐cleaning and recyclability of SERS substrates. ZnO, in particular, demonstrates higher photocatalytic and quantum efficiency compared to TiO₂, addressing the challenge of poor reproducibility in SERS substrates to some extent. Its anisotropic nature and high surface free energy promote strong binding with noble metals. Additionally, ZnO offers an efficient CT pathway, contributing to enhanced SERS sensitivity. Several studies have confirmed the exceptional performance of semiconductor‐metal heterojunction SERS substrates. For instance, Dang et al. synthesized double‐shelled ZnO, achieving a low limit of detection (LOD) of 1×10^−^⁷ M for 4‐MPY.^[14]^ Wang et al. developed ZnO/Ag bilayer structures on p‐type silicon wafers using photolithography and electrochemical methods, with a remarkable LOD of 10^−1^⁵ M for R6G and an enhancement factor (EF) estimated as 8.08×10^13^.^[15]^ Zhai et al. detected tyramine in beer by using Co‐doped ZnO modified with Au nanoparticles as a SERS substrate by achieving a LOD of 10^−^⁸ M.^[16]^ Similarly, Mohan et al. synthesized Ag/ZnO/Au 3D hybrid reusable SERS substrates, providing an ultra‐sensitive DNA detection platform and LOD of 10^−16^ M for R6G.^[17]^ However, easily synthesized, reusable, semiconductor‐bimetallic hybrid SERS substrates are less explored and worth immediate attention, which can detect even lower concentrations of analytes with higher SERS efficiency.

In this study, we present a comprehensive investigation of zinc oxide nanorods (ZnO NRs) modified with noble metals (Ag and Cu), proposed as cost‐effective, ultra‐sensitive and reusable SERS substrates. A conventional hydrothermal synthesis method was used for the synthesis of ZnO NRs, avoiding the use of complex techniques, which was subsequently applied for the preparation of all composite materials. We varied the composition of Ag and Cu in the composites to determine the optimum condition for highest SERS efficiency from the substrates. The SERS substrates were prepared by drop casting the alcoholic solution of the composites on a glass cover slip. With SERS EF of 6.2×10⁶, ZAC55 composites produced the best SERS performance for resonant Raman‐active analyte molecule R6G, adsorbed onto it. The same substrate showed trace level detection of R6G at very low concentrations such as 1aM (10^−18^ M), leading to observation of very strong IR peaks of R6G, which are generally not observed in Raman scattering. Furthermore, the photocatalytic degradation capability of the substrate was investigated using an appropriate theoretical model and reusability study was demonstrated.

## Experimental Details

### Materials

Zinc nitrate hexahydrate (Zn(NO₃)₂ ⋅ 6H₂O) and sodium hydroxide (NaOH) was purchased from SDFLC. Silver nitrate (AgNO₃), Rhodamine 6 G (R6G) and Methylene Blue (MB) were purchased from Sigma Aldrich. Sodium borohydride (NaBH_4_) was obtained from SD Fine Chem Ltd. Copper nitrate tetrahydrate was purchased from Finar. Polyvinylpyrrolidone (PVP) was purchased from SRL. Prior to usage, all glassware, coverslips, and silicon wafers were thoroughly cleaned using Milli‐Q water and acetone and subsequently dried.

### Preparation Methods

#### Preparation of ZnO Nanorods (NRs)

ZnO nanorods (NRs) were synthesized using a straightforward hydrothermal method. Initially, 0.2 M of Zn(NO₃)₂ ⋅ 6H₂O was dissolved in deionized water under continuous stirring to create a clear zinc precursor solution. Separately, a 0.8 M NaOH solution was prepared in deionized water. The NaOH solution was then added dropwise to the zinc nitrate solution while maintaining constant stirring, allowing NaOH to react with Zn^2+^ ions to form a zinc hydroxide intermediate. The mixture was stirred for an additional 20 minutes to ensure uniformity. The resulting solution was transferred into a Teflon‐lined stainless‐steel autoclave, sealed, and heated to 160 °C for 4 hours. After natural cooling to room temperature, the white precipitate of ZnO nanorods was collected by centrifugation and thoroughly washed with deionized water to remove any remaining ions. Finally, the nanorods were dried overnight at 60 °C.

#### Preparation of Metal Semiconductor Oxide Composite

The hybrid metal semiconductor oxide composites were synthesized through impregnation method, incorporating PVP as a stabilizing agent for silver nanoparticles. Initially, 0.03 M of earlier prepared ZnO NRs were added to the DI water, under constant stirring. Then 0.1 g of PVP was added into the prepared ZnO solution to act as a capping and stabilizing agent. Separately, a silver nitrate (AgNO₃)/ copper nitrate (Cu(NO_3_)_2_.3H_2_O) solution was prepared by dissolving 0.1 M of AgNO₃/ Cu (NO_3_)_2_.3H_2_O in deionized water. The corresponding solutions of noble metals were reduced to its metallic state by adding NaBH_4_ to the reaction mixture in a molar ratio of 2 : 1 (NaBH_4_ to metal ions). After that, the solution was quickly poured into the prior mixture under stirring. In order to ensure the homogeneous deposition, the reaction was allowed to continue at 90 °C for 1 hr under constant stirring. The reactants were allowed to be cooled to room temperature and were washed thoroughly with DI water once the reaction was finished. Then the grey precipitate was collected and dried in air for 6 hrs at 80 °C.

#### Preparation of Bimetallic Semiconductor Oxide Composite

The different composition of bimetallic semiconductor oxide was carried out using the same impregnation method as described for the metal semiconductor oxide by varying the composition of noble metals. In the following method instead of Ag/Cu both the noble metal's corresponding solutions were prepared and impregnated into the existing ZnO dispersion under continuous stirring. Then we followed the same procedure as for the synthesis of single metal semiconductor oxide. Different compositions were prepared by varying the concentration of noble metals while keeping the ZnO concentration constant for all the composition. The compositional variation of prepared composites is represented in Table 1. We synthesized a total of 6 composites (Figure S1, ESI) by varying the noble metals concentration. Comparative analysis of SERS performance was performed for all the composites. However, only five of these composites: ZA, ZAC 73, ZAC 55_,_ ZAC 37_,_ and ZC were selected for detailed characterization and analysis.

**Table 1:** Compositional variation of ZnO−Ag‐Cu (ZAC) composites by specifying the amount of silver nitrate and copper nitrate along with the consistent amount of ZnO.

Composite	ZnO content (mol)	Ag content (mol)	Cu content (mol)
ZA (ZnO−Ag)	0.03	0.1	–
ZAC 73 (ZnO−Ag−Cu)	0.03	0.07	0.03
ZAC 64 (ZnO−Ag−Cu)	0.03	0.06	0.04
ZAC 55 (ZnO−Ag−Cu)	0.03	0.05	0.05
ZAC 37 (ZnO−Ag−Cu)	0.03	0.03	0.07
ZC (ZnO−Cu)	0.03	–	0.1

### Characterization

The structural characterization of the composites was performed using X‐ray diffraction (XRD) patterns obtained with a Rigaku Ultima IV X‐ray diffractometer, employing Cu Kα radiation (λ=1.54 Å). The elemental composition was determined through energy‐dispersive X‐ray fluorescence (ED‐XRF, PANalytical, epsilon 1). A field emission scanning electron microscope (FE‐SEM, FEI Apreo) was used to examine the morphological features. Surface composition analysis was performed using Thermo Scientific K‐α X‐ray photoelectron spectroscopy (XPS) with aluminium Kα radiation (1486.6 eV). The extinction spectra of the composites were recorded with a Jasco V‐670 UV‐visible spectrophotometer. The solid‐state photoluminescence (PL) spectra of the substrates were measured using a Horiba Fluorolog spectrofluorometer. Both the excitation and emission slit widths were maintained at 6 nm during data acquisition. Horiba Delta flex Modular fluorescence lifetime system was used for all lifetime measurements. Instrumental parameters for lifetime measurement were set as follows: LED excitation source‐377 nm Nano LED, Peak preset‐10,000 counts.

### SERS Substrate Preparation

A small amount of the prepared composites was dispersed in alcohol and the solution was sonicated for 10 mins to ensure the uniform deposition of added particles, as they were subsequently drop cast on to the glass coverslips with 18 mm ×18 mm dimension to be used as the SERS substrates. The coverslips were thoroughly cleaned by acetone and DI water, before use. The SERS performance and photocatalytic degradation of prepared composites was studied by using a common analyte molecule R6G. First, we prepared the 10^−4^ M stock solution of R6G in methanol. Then from the stock solution in methanol, a series of lower concentrations down to 10^−18^ M were prepared by successful dilution. Subsequently, 40 μL methanolic solution of R6G was drop cast on the prepared SERS substrates and allowed to dry for 10–15 mins under ambient condition before the collection of SERS spectra. A similar procedure was also followed for MB detection.

### SERS Measurement

SERS measurements were carried out by using a UniRAM‐3300 micro‐Raman mapping spectrophotometer. A continuous‐wave diode‐pumped solid‐state laser operating at λ=532 nm was used to record the Raman spectra of the R6G adsorbed composites, with a constant laser power of 124 μW and an acquisition time of 30 seconds. A 50x air‐immersion objective lens (NA=0.55) was used to focus the laser on the sample surface and collect the backscattered light. The system was calibrated using naphthalene as a reference. The Raman signal was detected by a highly sensitive TE‐cooled charge‐coupled device (CCD) linear array detector (2000×256 pixels) connected to a spectrograph. Data acquisition and processing were controlled using Andor Solis software.

## 2 Results and Discussion

### 2.1 X‐Ray Diffraction (XRD) Analysis of the Composites

**Figure 1:**
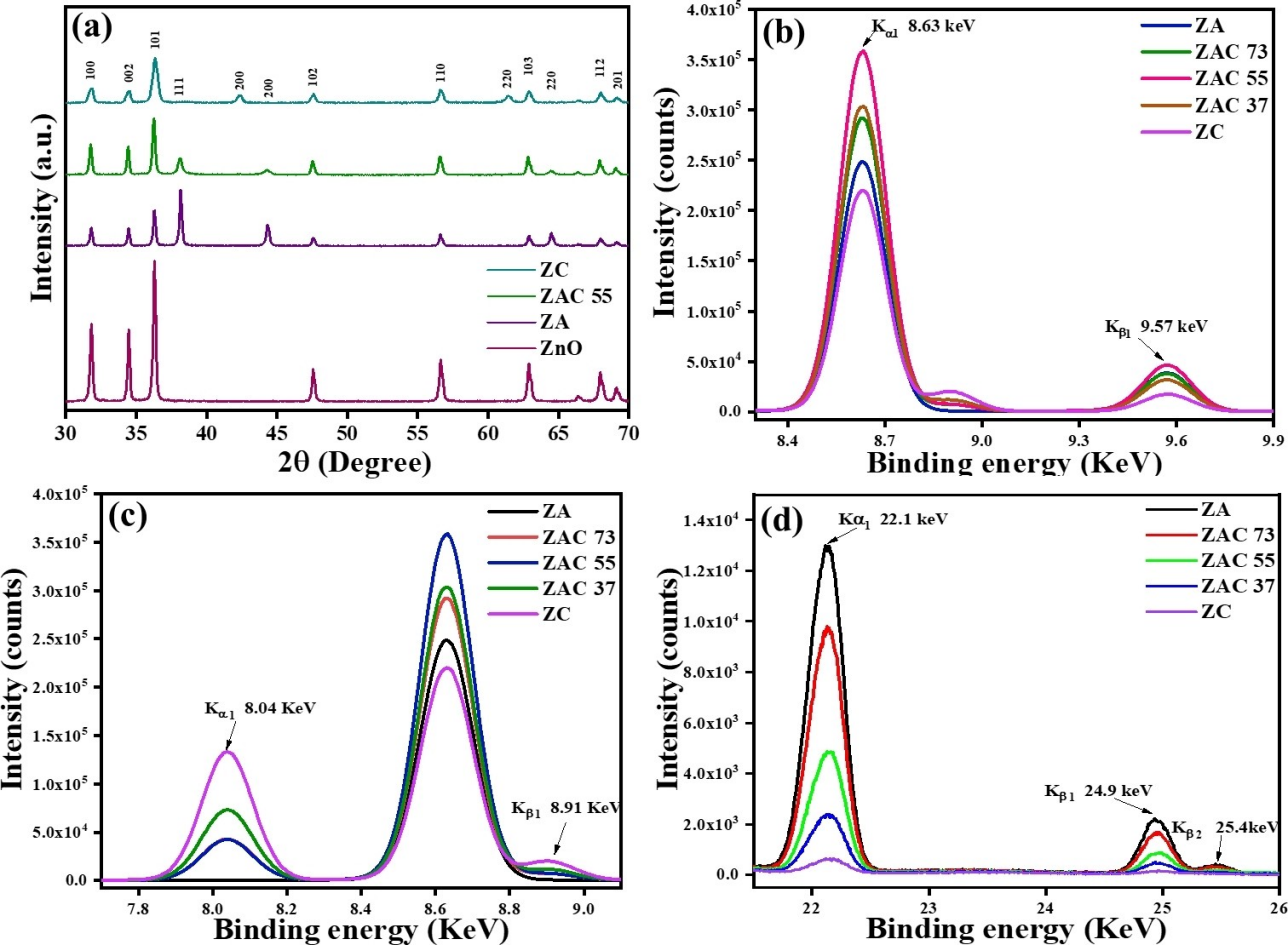
(a) XRD patterns of ZnO and different composites: ZA, ZAC 55 and ZC. ED‐XRF spectra of composites, showing the peaks for: (b) Zn, (c) Cu and (d) Ag.

The crystallographic structure of the prepared composites was studied by using X‐ray diffraction (XRD) technique with an incident angle of two degree, as shown in Figure 1a. The XRD pattern revealed several prominent peaks corresponding to different phases, present in the composite.^[18]^ The diffraction peaks of ZnO NRs were ascribed to (100), (002), (101), (102), (110), (103), (112) and (201) crystal planes of wurtzite hexagonal structure of ZnO which matched well with JCPDS#36–1451 observed at 31.81, 34.47, 36.29, 47.57, 56.62, 62.88, 67.95 and 69.1 in 2θ degree^[19]^ and the sharpness of the peaks suggested a high degree of crystallinity. The evolution of additional peaks in ZAC 55 was due to presence of Ag and Cu in the composition. The additional diffraction peaks for Ag, observed at 38.1, 44.3, 64.4 (in 2θ) were indexed to (111), (200), (220) planes of face centred cubic (fcc) structure of Ag (JCPDS#04–0783) appeared as silver was in elemental form,^[20]^ without any observable formation of silver oxides. For Cu, observed diffraction peaks at 42.3, 61.4 were ascribed to (200), (220) crystallographic plane (JCPDS#78–2076), indicating the incorporation of cuprous oxide (Cu_2_O) instead of Cu into the composite.^[21]^ Thus, the PXRD result showed that the composite consisted of ZnO, Ag and Cu_2_O phases, without formation of new phases. The presence of distinct and well‐defined peaks for each material indicated that there was no significant structural deformation during composite synthesis. The intensity of peaks in ZAC, corresponding to the ZnO phase was higher than those for Ag and Cu_2_O, indicating that ZnO was the dominant phase in the composite. The intensity of Cu_2_O (200), (220) peaks was found to be very low, but on magnification the peaks coincided with ZC peaks (Figure S2, ESI).

### 2.2 X‐Ray Fluorescence (XRF) Studies of the Composites

X‐ray fluorescence (XRF) analysis was performed to confirm the elemental composition of the synthesized composites. The distinct peaks in XRF spectra (Figures 1b–d) indicated the presence of Zn, Cu and Ag as the main elements in the respective composites, with no significant impurities detected. The observed peaks K_α1_ and K_β1_ at 8.63 KeV and 9.57 KeV ensured the presence of Zn in the all‐prepared composites^[22]^ (Figure 1b). The presence of Cu was confirmed due to the presence of the peaks at 8.04 KeV and 8.91 Kev^[23]^ (Figure 1c). The XRF peaks K_α1_, K_β1_ and K_β2_ at 22.1 KeV, 24.9 KeV and 25.4 Kev, respectively,^[24]^ represented the presence of Ag in the composites (Figure 1d). The Ag and Cu elemental compositions obtained from ED‐XRF are presented in Table 2, which demonstrates a good correlation between the input and resulting ratios. With the decrease in concentration of Cu and Ag in the composites, a systematic decrease in the intensity of the peaks was demonstrated (Figure 1c, d).

**Table 2:** Elemental composition of Ag, Cu and Zn obtained through ED‐XRF.

Composite	Composition		
	Zn	Ag	Cu
ZA	58.4 %	41.5 %	–
ZAC 73	60.3 %	27.9 %	11.7 %
ZAC 55	77.3 %	11.6 %	11.1 %
ZAC 37	70.5 %	7.6 %	21.8 %
ZC	56.0 %	–	43.9 %

### 2.3 FE‐SEM Study of the Composites

**Figure 2:**
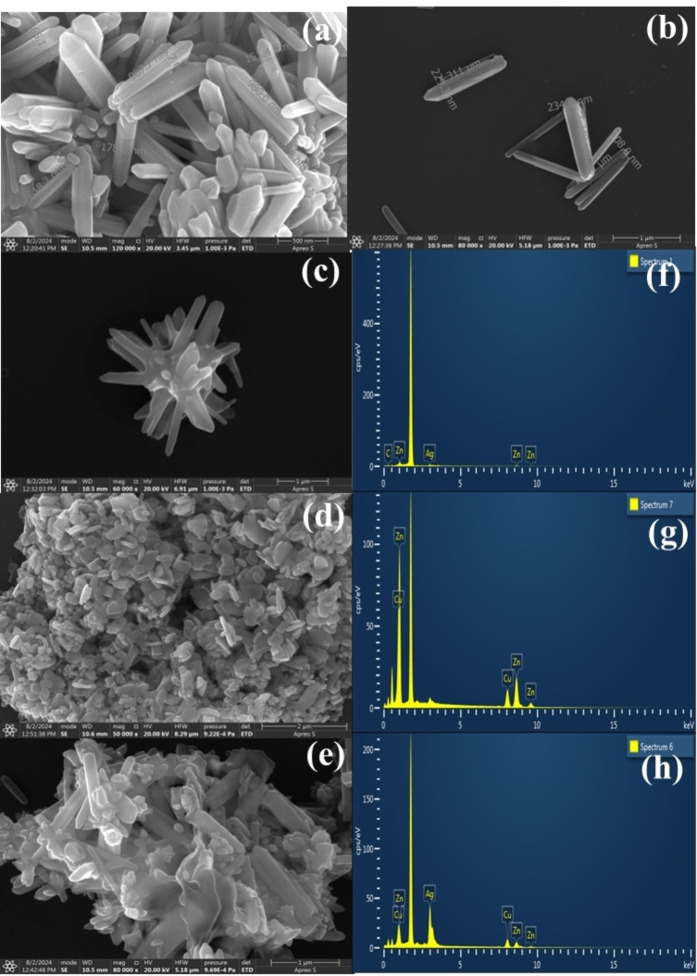
FE‐SEM images of prepared composites: (a‐b) ZnO, (c) ZA, (d) ZC, (e) ZAC 55. In the respective rows, the corresponding EDS spectra of ZA(f), ZC(g) and ZAC(h) are shown.

To examine the morphology of the synthesized composites, field emission scanning electron microscopy (FE‐SEM) was utilized. Figure 2 depicts the FE‐SEM images of all the prepared composites along with bare ZnO. The ZnO appeared as rod‐like with diameter in the range of 52–367 nm and length of 0.5–2 μm (Figure 2a, b). During the hydrothermal synthesis, zinc precursor (Zn^2+^) reacted with sodium hydroxide (OH^−^) to form Zn(OH)_2_, which was converted to ZnO at elevated temperature and pressure. The anisotropic wurtzite structure of zinc oxide allowed it to form as rods due to the growth in c‐axis.^[25]^ These structures provided high surface area, which was advantageous for enhancing the surface related phenomena such as adsorption and photocatalytic reaction. As can be compared between Figure 2c and d, for ZA, the Ag nanoparticles were dispersed across the surface of ZnO, indicating a good distribution of Ag within the composite matrix as no isolated NPs were observed. The close proximity of Ag NPs, is critical for SERS, as it leads to the formation of “hot spots” where the electromagnetic enhancement is maximized. However, Figure 2d confirmed that the Cu_2_O particles appeared as nearly spherical, isolated structures in ZC, not as dispersed as Ag. The introduction of copper tends to form larger spherical particles because of its different reduction potential and growth mechanism. Copper ions might preferentially aggregate and nucleate in localized regions, leading to the growth of larger spherical structures instead of uniformly dispersing like silver.[^26^, ^27^] Figure 2e revealed a heterogenous mixture of ZnO nanostructure, Ag and Cu_2_O particles for ZAC 55. Similar morphology was also obtained for ZAC 37 and ZAC 73 (Figure S12, ESI). The incorporation of noble metals into the ZnO NRs did not specifically change the morphology of ZnO but probably enhanced the surface roughness. The composition of these nanostructures achieved high surface area and heterogeneous interface which was essential for CM enhancement in SERS.

### 2.4 XPS Analysis of the Composites

**Figure 3:**
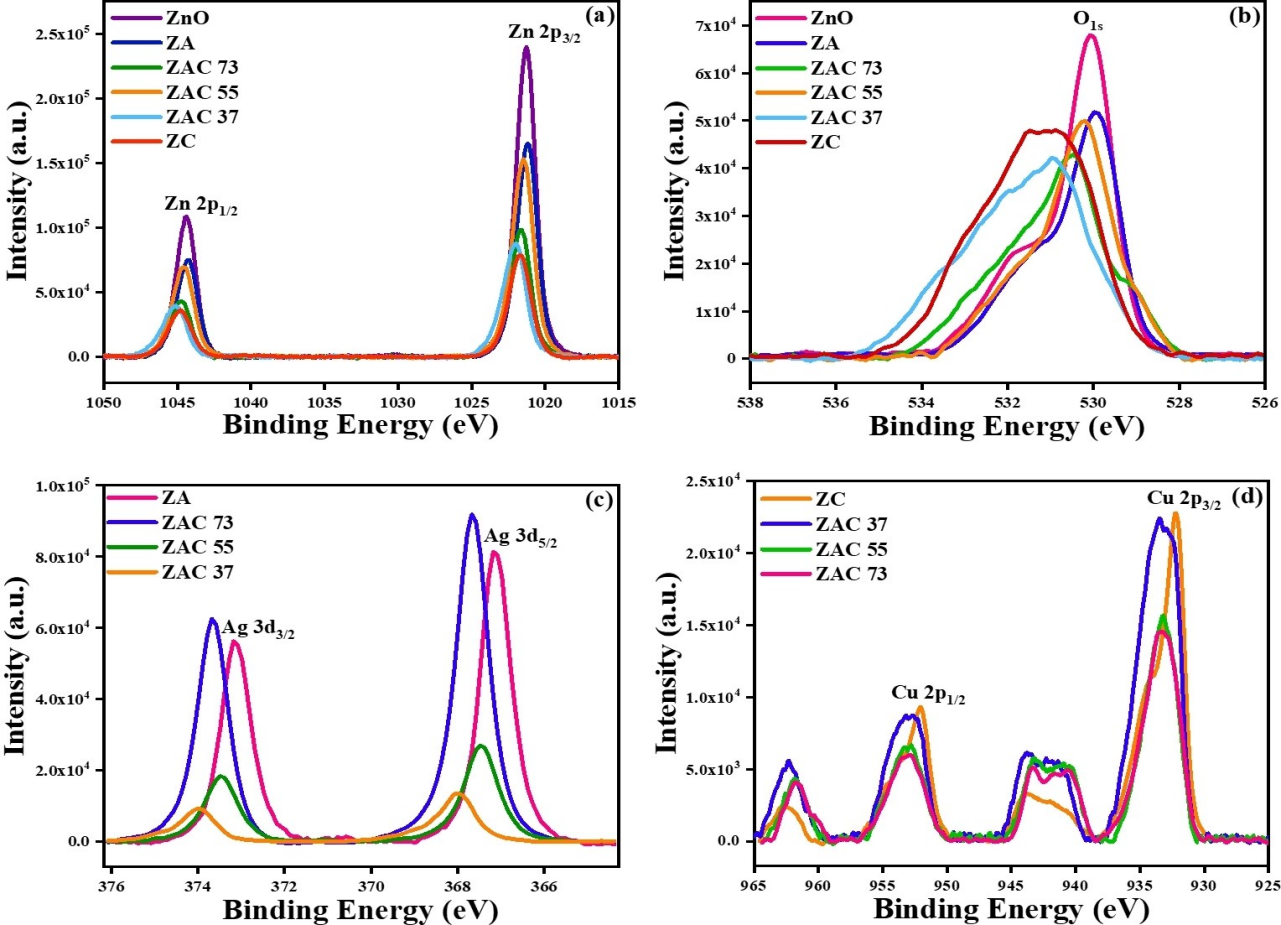
High resolution XPS spectra of all the synthesized composites for the selective elements: (a) Zn2p (b) O1s (c) Ag3d (d) Cu2p.

The oxidation states and the surface chemical composition of the constituent elements present in the composites were investigated from XPS spectra.^[28]^ The survey spectrum (Figure S3a, ESI) confirmed the presence of Zn, O, Ag and Cu elements along with minor peaks corresponding to carbon (C1s) attributed to residual organic impurities or atmospheric adsorption during sample handling indicating successful formation of the composite material. The high‐resolution XPS spectra of Zn2p in ZnO showed two prominent peaks at binding energies of approximately 1021.2 eV and 1044.3 eV, corresponding to the Zn 2p₃/₂ and Zn 2p₁/₂ states (Figure 3a). These peaks are characteristic of Zn^2+^ in the ZnO lattice, confirming that zinc exists predominantly in the oxidized form. The binding energy separation of 23.1 eV between the Zn 2p₃/₂ and Zn 2p₁/₂ peaks was consistent with the values reported for ZnO for all the prepared composites, indicating the successful formation of ZnO in the composite.^[29]^ The O1s spectra exhibited only one peak, however with some variations in the peak positions among different composites (Figure 3b). The peak at 530 eV was attributed to lattice oxygen in the ZnO matrix.^[29]^ The evolution of same peak confirms the presence of O1s in the composite ZA at same oxidation state, which ensures that with the addition of Ag there is no further oxidation occurred in the ZA composite. Except ZA composite, O1s peak of all the other composites were found to be in higher binding energy region confirming that they were in higher oxidation state. These oxygen species played a crucial role in enhancing the surface reactivity of the composite and contributed to the catalytic properties of the material. The Ag 3d spectrum (Figure 3c) of ZA revealed two peaks at binding energies of 367.1 eV (Ag 3 d₅/₂) and 373.1 eV (Ag 3 d₃/₂),^[29]^ with a spin‐orbit splitting of 6.0 eV. These values were consistent with metallic silver (Ag⁰), confirming the reduction of silver nitrate (AgNO₃) to elemental silver nanoparticles during the synthesis. This indicated the absence of oxidized silver species (Ag^+^), suggesting that the silver remained in its metallic state in ZA composite, which was essential for enhancing the plasmonic properties of the composite for applications such as SERS. However, Ag was partially oxidised for all the bimetallic composites, although binding energies of the Ag 3d electrons in the composites were not significantly higher compared to ZA. Among the composites, Ag 3 d electrons in ZAC 55 showed lowest binding energy compared to ZAC 73 and ZAC 37 (Figure 3c), indicating that the ZAC 55 composite was least oxidised among all the bimetallic composites, enabling it as a good substrate for SERS measurement. The Cu 2p spectrum of ZC (Figure 3d) exhibited two main peaks at binding energies of 932.1 eV (Cu 2p₃/₂) and 952 eV (Cu 2p₁/₂), which confirmed the presence of Cu^+^ as Cu₂O. However, the appearance of a satellite feature between 940 eV–943 eV also suggested the presence of Cu^2+^. This satellite peak is a well‐known feature in the XPS spectra of Cu^2+^ in CuO compounds.^[29]^ The co‐existence of both Cu₂O and CuO provided a broader range of redox activity and might enhanced the composites’ performance in photocatalysis or other catalytic applications by improving charge separation and increasing reactive sites.

### 2.5 Absorption Spectra and Band‐Gap Calculations of the Composites

**Figure 4:**
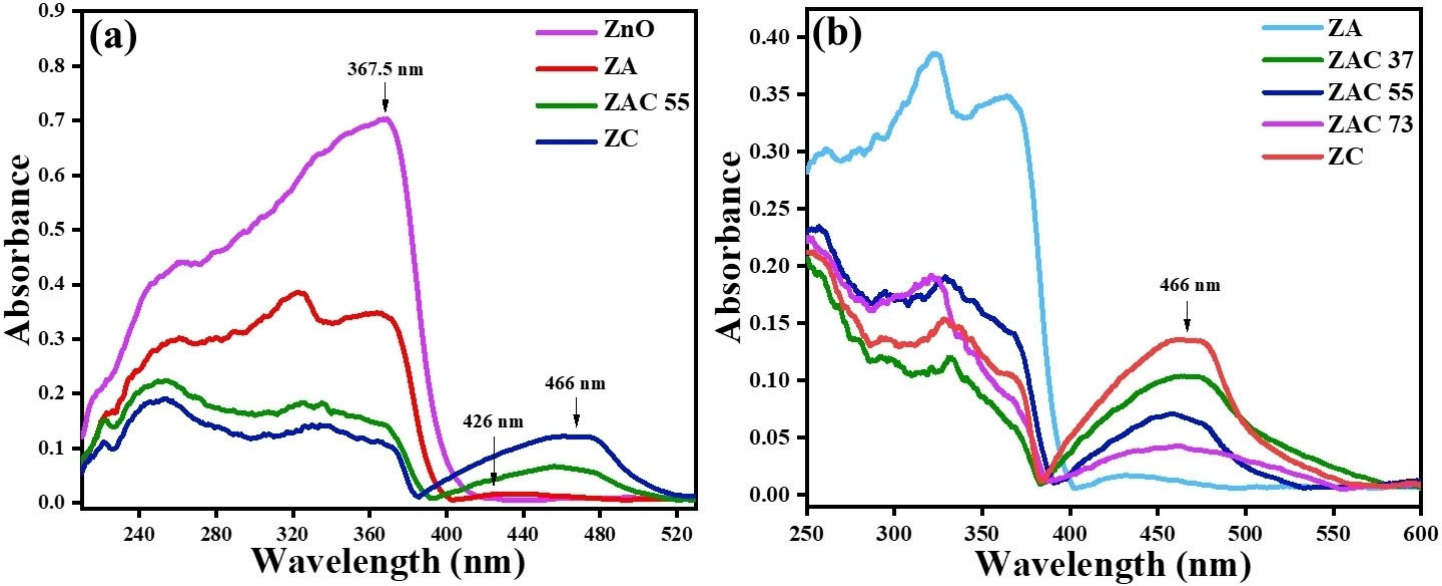
Absorption spectra of (a) different composites and only ZnO; (b) all the composition of ZAC with ZA and ZC.

Figure 4a illustrated the optical absorption spectra of all ZnO composites with noble metals (Ag and Cu) in comparison to bare ZnO NRs. When ZnO was treated with Ag or Cu, plasmonic metal atoms replaced Zn atoms within the ZnO crystal lattice, introducing impurities into the structure and altering its electronic and optical properties. These changes affected the material's absorption coefficient, band gap, extinction coefficient, and transparency. It was observed that bare ZnO exhibited the highest absorbance but only in the UV range, with no absorbance in the visible region. However, upon impregnated with Ag and Cu, an additional absorption band showed up into the higher (visible) wavelength region. ZnO NRs demonstrated a broad absorption peak, with a maximum at 367 nm, confirming the material's purity. Upon treated with Ag, the additional plasmonic peak showed up at 426 nm for ZA, while Cu in ZC composite, resulted in an absorbance peak at 466 nm. In the visible, the highest and lowest absorption were found in ZC and ZnO, respectively,^[30]^ while ZAC showed absorption between ZC and ZA. Additionally, the extinction spectra of the composites revealed blue‐shift for the plasmonic peak compared to ZC, as the silver concentration increased (Figure 4b and S4, ESI).

The band gap energy of the prepared ZnO samples was estimated using Tauc's method. A plot of (αhν)^2^ versus hν, was used to determine the band gap. The variation of (αhν)^2^ as a function of energy of the photons was studied according to Tauc model: (αhν)^2^=A(hν‐E_g_), where α is the absorption coefficient, h is the Planck constant, ν is the photon frequency, A is a constant, E_g_ is the band gap. The bandgap energy (E_g_) was obtained by extrapolating the linear portion of the curve to the x‐axis.^[31]^ The estimated E_g_ values for ZnO, ZA, ZAC 73, ZAC 55, ZAC 37, ZC were found to be 3.18 eV, 3.17 eV, 3.06 eV, 2.50 eV, 2.43 Ev and 2.49 eV respectively, as shown in Figure S5, ESI. A clear decrease in bandgap values was observed when ZnO was impregnated with Cu (ZC), compared to ZA. The presence of Cu^2+^ ions in the composite created energy levels within the ZnO bandgap, enhancing visible light absorption. This effect was further imputed to the formation of p‐n heterojunctions between ZnO (an n‐type semiconductor) and Cu₂O (a p‐type semiconductor), which promoted efficient charge separation and reduced recombination rates of photogenerated charge carriers beneficial for SERS and photocatalytic applications. Most importantly for us, the bandgap of bimetallic semiconductor oxide ZAC55 was found to be smaller (2.50 eV), closer to ZC bandgap, ensuring efficient photoexcitation of the electrons.

### 2.6 SERS Studies

#### 2.6.1 Comparative Analysis of SERS Performance of Different Composites

**Figure 5:**
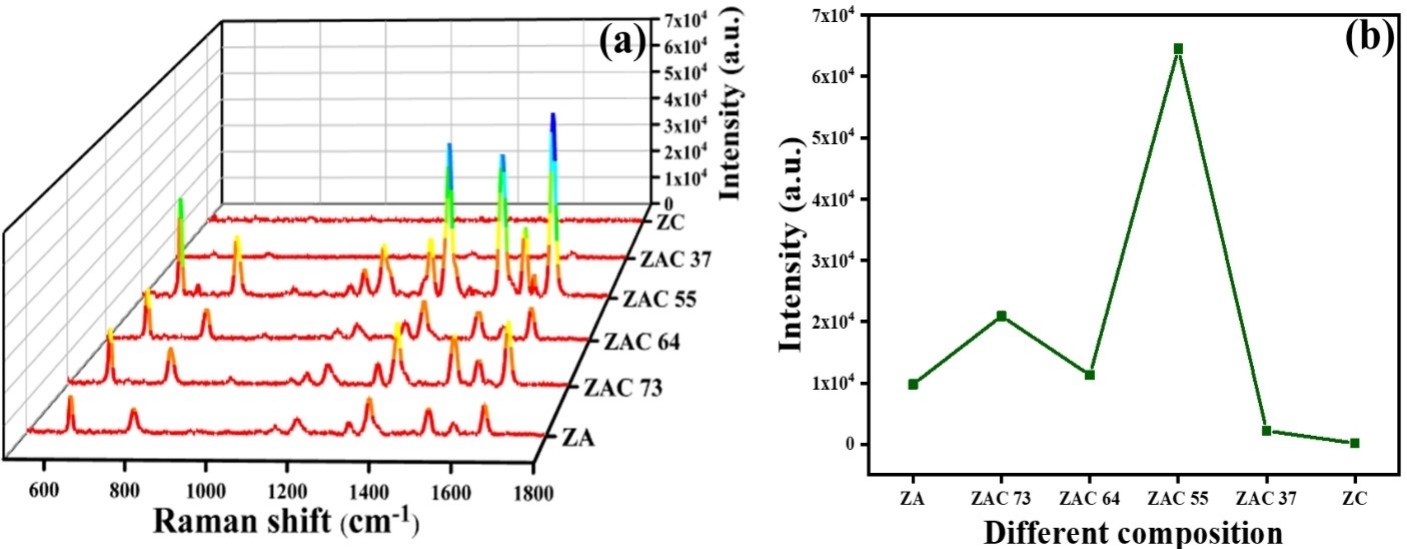
(a) SERS spectra of 10^−8^ M R6G adsorbed on all the prepared compositions (ZA, ZAC, ZC); (b) Comparison of 1653 cm^−1^ peak from the SERS spectra displayed in (a) for all the substrates. All spectra are obtained with 1 s exposure time along with 30 accumulations, after excitation with a 532 nm laser with average power 124 μW.

To study the comparative SERS performance of the composites, R6G was used as the analyte molecule, which was a resonant scatterer at our excitation wavelength of 532 nm. 10 nM R6G solution (in methanol) was drop cast on each of the substrates, ZA, ZAC 73, ZAC 64, ZAC 55, ZAC 37 and ZC, followed by an excitation with laser light. Figure 5a shows the SERS spectra of R6G, collected from different substrates. ZAC 55 stood out as the best substrate as it produced maximum SERS intensity, when a representative peak at 1653 cm^−1^ of R6G was considered for comparison (Figure 5b). ZAC 55 produced approximately 6~fold higher signal than ZA. In a blank study, SERS spectra of the ZAC55 substrate without R6G did not produce any characteristic Raman peak (Fig S7, ESI). Also, negligibly small SERS intensity was recorded with ZC, probably due to lack of interaction between ZnO and Cu, and easy oxidation of Cu to lose the plasmonic effect substantially. ZAC 55 substrate's SERS enhancement factor (EF) was determined using 1,2,3‐benzotriazole (BTA), a non‐resonant and nonfluorescent reporter molecule, instead of R6G. The strong fluorescence of R6G, hindered the collection of Raman signals under our experimental conditions. The EF for ZAC 55 was calculated as 6.2×10⁶ (Figure S8 and Calculation S1, ESI) using the equation EF=(I_SERS_/I_Raman_) × (N_Bulk_/N_Surface_), where I_SERS_ and I_Raman_ represent the signal intensities in the SERS and normal Raman spectra of BTA, respectively. N_Bulk_ and N_Surface_ refer to the number of analyte molecules excited by the laser in bulk Raman and SERS experiments, respectively.^[32]^

#### 2.6.2 Evaluating Heterogeneity and Density of Hot Spots Using Raman Mapping Study

**Figure 6:**
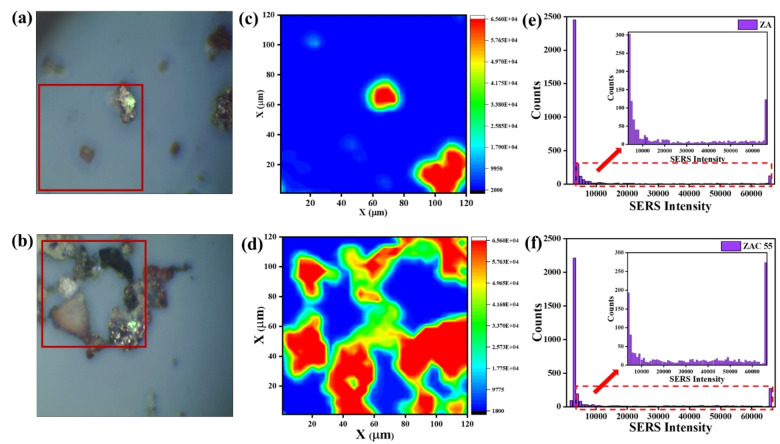
Optical images collected under Raman microscope, using 50X Objective for (a) ZA (b) ZAC 55 substrate after drop cast; Raman mapping image of 10^−4^ M R6G adsorbed on the (c) ZA and (d) ZAC 55 substrate, where the SERS mapping was performed by considering the peak at 1577 cm^−1^; (e, f) represent histogram of SERS counts for c and d, respectively. The inset in each histogram plot, shows the counts obtained only from the marked area shown in (a, b).

Raman mapping study was performed for ZAC55 and ZA to assess and compare the distribution and density of the hotspots within the substrate. The main objective of the analysis was to study the heterogeneity of the surface of the SERS substrate. The experiment was performed by adsorbing 10^−4^ M R6G on the substrate and scanning over a large surface area (120 μ m ×120 μ m) with a step size of 2 μm, which constituted 3600 detection points. Figures 6a, b shows the optical images of the substrate ZA and ZAC 55, respectively, with the marked region indicating the specific area selected for the mapping study. By employing the intensity of a representative Raman band at 1577 cm^−1^ for R6G, Raman mapping (Figure 6c, d) highlighted the distinct region with maximum intensities, indicating the hotspots for both ZA and ZAC 55. Figures 6e, f represent histogram of SERS counts for ZA and ZAC 55, respectively, obtained from the mapping data (6c, d). It is clearly seen that the density of hotspots significantly increases in ZAC 55 with more counts with higher SERS intensities, compared to ZA.

Moreover, the variation in randomly collected SERS signal intensities obtained from ZAC55 substrates (Figure S9, ESI), corroborated well with the result obtained from mapping data, clearly suggesting that the hotspots were heterogeneously distributed on the substrate. For instance, the bars with higher intensity (Figure S9a, ESI) represented the bright regions in the mapping data (Figure 6d), indicating the hot spots, while minimum or absence of the hotspots resulted into a lower intensity. We propose, these hotspots are primarily associated with regions where Ag nanoparticles are closely spaced in the composite along with ZnO and Cu (Figure S6, ESI), creating strong localized electromagnetic fields that significantly enhance the Raman scattering of nearby molecules.

#### 2.6.3 SERS‐Based Detection of R6G and MB at Ultralow Concentrations

**Figure 7:**
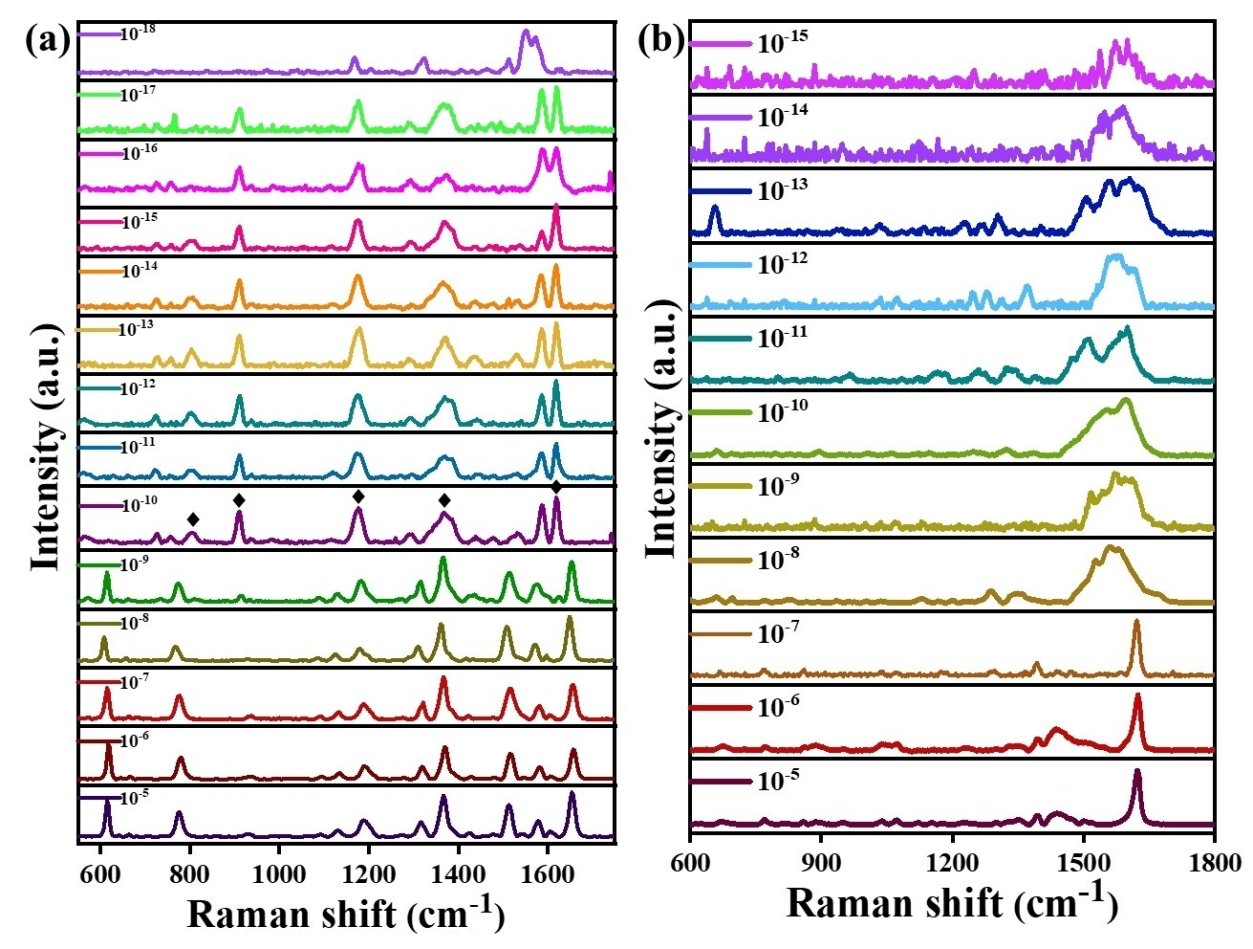
(a) Attomolar level SERS‐based detection of R6G by using ZAC 55. The symbol ‘♦’ in 10^−10^ M R6G SERS spectra marks the strong IR peaks, which are otherwise absent in SERS spectra at higher concentrations of R6G, i.e till 10^−9^ M, (b) Femtomolar level SERS‐based detection of MB by using ZAC 55.

Subsequently, to assess the SERS efficacy of the substrates, we evaluated the LOD for R6G using all the composites ZA, ZAC 73, ZAC 55, ZAC 37 and ZC, by measuring the SERS spectra of R6G. While the LOD of R6G detection for ZA, ZAC 73 and ZAC 37 was found to be 10^−12^ M, 10^−11^ M, and 10^−9^ M, respectively (Figure S11, ESI), ZAC55 unveiled clear and resolved characteristic peaks of R6G, till at an ultralow 10^−18^ M concentration (Figure 7a), demonstrating the unprecedented SERS efficiency of ZAC55 composite over other substrates. The ZC composite did not show any SERS signal of R6G even at higher concentration. The probable reason could be that Cu was completely oxidised, resulting into loss of its plasmonic efficiency. Moreover, the universality of the sensor was confirmed by performing a similar study with a different analyte molecule Methylene Blue (MB), which was non‐resonant at 532 nm excitation. The obtained LOD (10^−15^ M), best till date for MB via SERS method, also proved the supreme efficacy of ZAC55 substrate (Figure 7b). The unprecedented SERS efficiency of ZAC55 was a result of the synergistic effect between ZnO, Ag, and Cu₂O. ZnO NRs provided a high surface area for the dispersion of Ag nanoparticles and acted as scaffolds that facilitated the uniform distribution of hotspots. Ag nanoparticles provided the plasmonic enhancement. Whereas, the presence of Cu₂O enhanced visible‐light absorption and contributed additional charge transfer pathways (*Vide infra*), which further enhanced the signal through chemical enhancement mechanisms. Infact, ZAC 55 was found to be best SERS‐based sensor in terms of LOD for R6G and MB, among similar other plasmonic substrates with ZnO found in literature (Table 3).

**Table 3:** A comparison of SERS‐based LOD values for different analyte molecules including R6G and MB, using different SERS substrates including metal‐ZnO heterostructures.

Substrates	Probe molecule	EF	LOD (excitation source)	Ref
coralloid ZnO@Ag	R6G, MG	1.61×10^7^	10^−12^ M, 10^‐9^ M(532 nm laser)	[35]
Ag/Au/ZnO/P	4‐MBA, Thiram	7.09×10^7^	10^−11^ M, 10^−11^ M (785 nm laser)	[36]
Si‐based ZnO/Ag bilayer	R6G	8.08×10^13^	10^−15^ M (532 nm laser)	[15]
ZnO/Ag Sea urchin	R6G	2.98×10^6^	10^−16^ M (514.5 nm laser)	[37]
Doubled shelled ZnO	4‐MPY	1.2×10^4^	10^−7^ M (532 nm laser)	[14]
Ag/ZnO/Au 3D hybrid	R6G	1.0×10^10^	10^−16^ M (488 nm laser)	[17]
Patterned ZnO NR array with Ag	R6G	1.80×10^10^	10^−13^ M (532 nm laser)	[38]
Ag‐fZnO NRs	R6G	2.43×10^8^	10^−9^ M (532 nm laser)	[39]
Al/ZnO/ZIF‐M	CV	1.93×10^6^	2.81×10^−11^ M (532 nm laser)	[40]
Au functionalised ZnOTP	apomorphine	7×10^6^	1 μM (633 nm laser)	[41]
3D Ag NPs decorated ZnO/ Si heterostructure	R6G, melamine	8.7×10^7^	10^−16^ M, 10^−10^ M (633 nm laser)	[42]
ZnONR@AgND	R6G	3.3×10^9^	10^−12^ M (532 nm laser)	[43]
Au/Bi_2_O_3_	MB, MO	9.22×10^6^	10^−11^ M, 10^−9^ M (532 nm)	[44]
Paper based silver and gold NPs	MG, MB, CV	9.0 × 10^7^	10^−9^ M, 10^−8^ M, 10^−8^ M	[45]
Au NPs on capillary substrate	R6G, CV	1.0×10^7^	10^−11^ M, 10^−10^ M (532 nm, 784 nm laser)	[46]
Fe_3_O_4_@CoNiLDH@Ag NP	MB	5.81×10^8^	10^−10^ M (633 nm)	[47]
MPA/AgNP 3D‐SERS substrate	R6G, thiram	8.8×10^9^	10^−12^ M, 10^−7^ M	[48]
Ag NPs on ZnO plates	MB	6.2×10^6^	10^−9^ M	[49]
ZAC 55 heterostructure	R6G, MB	6.2×10^6^	10^−18^ M, 10^−15^ M (532 nm laser)	this work

One important point to note here is that, after nM concentration, we observed very different SERS spectra for R6G, with disappearance of few intense peaks and intensification of some other non‐existing peaks (Figure 7a). The probable reason could be, at low concentrations, starting from 10^−10^ M onwards, R6G molecules preferentially oriented themselves in a specific and different way for the adsorption on the surface. This change in orientation could enhance specific vibrational modes of the molecule which were very strong IR bands otherwise, but remained absent in Raman.[^33^, ^34^] The detailed description of the assignment of R6G and MB vibrational peaks are provided in Table S2 and S3, ESI, respectively. For example, a very strong IR peak at 1607 cm^−1^ due to skeletal stretching vibration of the external phenyl mode of R6G, remained as a very weak shoulder peak for R6G at our experimental condition till nM concentration, but reappeared with high intensity thereafter. Thus, all the hitherto known strong IR bands (indicated in Figure 7a), which appeared in the SERS spectra of R6G after nM concentration, must be result of a different pattern of adsorption and subsequent change in polarizability of the corresponding vibration.

### 2.7 Photoluminescence Spectra and Lifetime Measurements of the Composites

**Figure 8:**
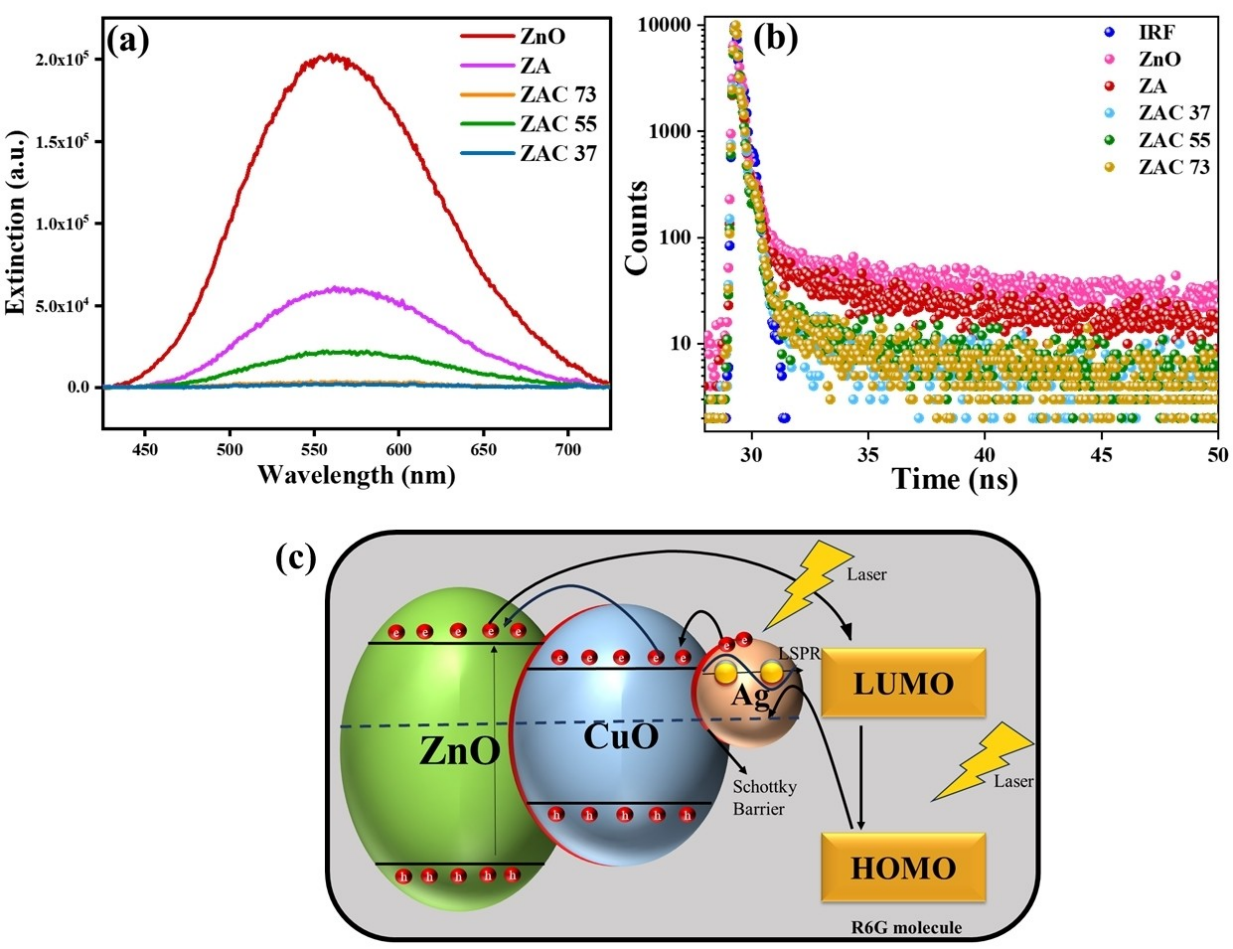
(a) Photoluminescence spectra and (b) time‐resolved photoluminescence (TRPL) decay of ZnO and the composites; (c) A schematic of the charge transfer mechanism in ZAC composite for the detection of R6G molecule.

Photoluminescence (PL) spectra of the composites provide critical insights into the behaviour of photogenerated charge carriers and their recombination dynamics.^[50]^ With 350 nm excitation, the defect band emission of ZnO displayed a broad characteristic peak centred at 560 nm, with the PL intensity of the peak progressively decreasing from ZnO to composites in the following order: ZAC37, ZAC73<ZAC55<ZA<ZnO (Figure 8a). This reduction in PL intensity suggests an improved charge transfer mechanism within the composite systems. The incorporation of Ag and Cu introduces additional electronic states at the interfaces, which act as electron traps. These states enhance the separation of photogenerated charge carriers by capturing electrons from ZnO, thereby reducing recombination. Thus, the lower PL intensity indicates more efficient charge transfer, which significantly contributes to improved SERS performance and photocatalytic activity (*vide supra*). The decay profiles of the PL emission of ZnO and the composites (Figure 8b) supports the trend observed in Figure 8a, as the excited state lifetimes of the ZAC composites were found to be much shorter than pure ZnO (Table S1, ESI). It is to be noted here that although ZAC 37 and ZAC 73 have shown better charge transfer efficiency compared to ZAC 55, their SERS performance is poor compared to ZAC 55. This could be due to the higher oxidation of the Ag in ZAC37 and ZAC73 compared to Ag in ZAC55, as evident from the XPS data in Figure 3c, reducing the plasmonic efficiency of ZAC37 and ZAC73.

The underlying charge transfer mechanism^[11]^ of ZAC substrates is explained as follows. As shown in Figure 8c, under laser excitation, Ag nanoparticles generate a LSPR effect, injecting electrons into the conduction band (CB) of CuO. The electrons are then transferred to the CB of ZnO due to the favourable alignment and subsequently to the lowest unoccupied molecular orbital (LUMO) of the R6G molecule. The semiconductor heterojunction (ZnO−CuO) facilitates this charge transfer between Ag and R6G molecules, a process that is not as effective in the Ag/R6G system alone. Additionally, the formation of Schottky junctions at the Ag/ZnO and Ag/CuO interfaces redistributes electrons creating strong electric filed, further amplifying the SERS signal due to enhanced charge transfer. In another pathway, electrons are excited directly from the valence band (VB) of ZnO to its CB under laser irradiation and follows the same transfer route as the first pathway. A third pathway involves laser‐induced electron excitation from the highest occupied molecular orbital (HOMO) of R6G to the Fermi level of Ag. These electrons are then transferred to the CB of CuO, followed by the CB of ZnO, and finally back‐excited to the LUMO of R6G, with eventual transition to the HOMO of R6G. This sequential energy transfer facilitates efficient charge separation, suppressing electron‐hole recombination and enhancing the contribution of charge transfer to the overall SERS signal.

### 2.8 Photocatalytic Degradation Process and its Mechanism

**Figure 9:**
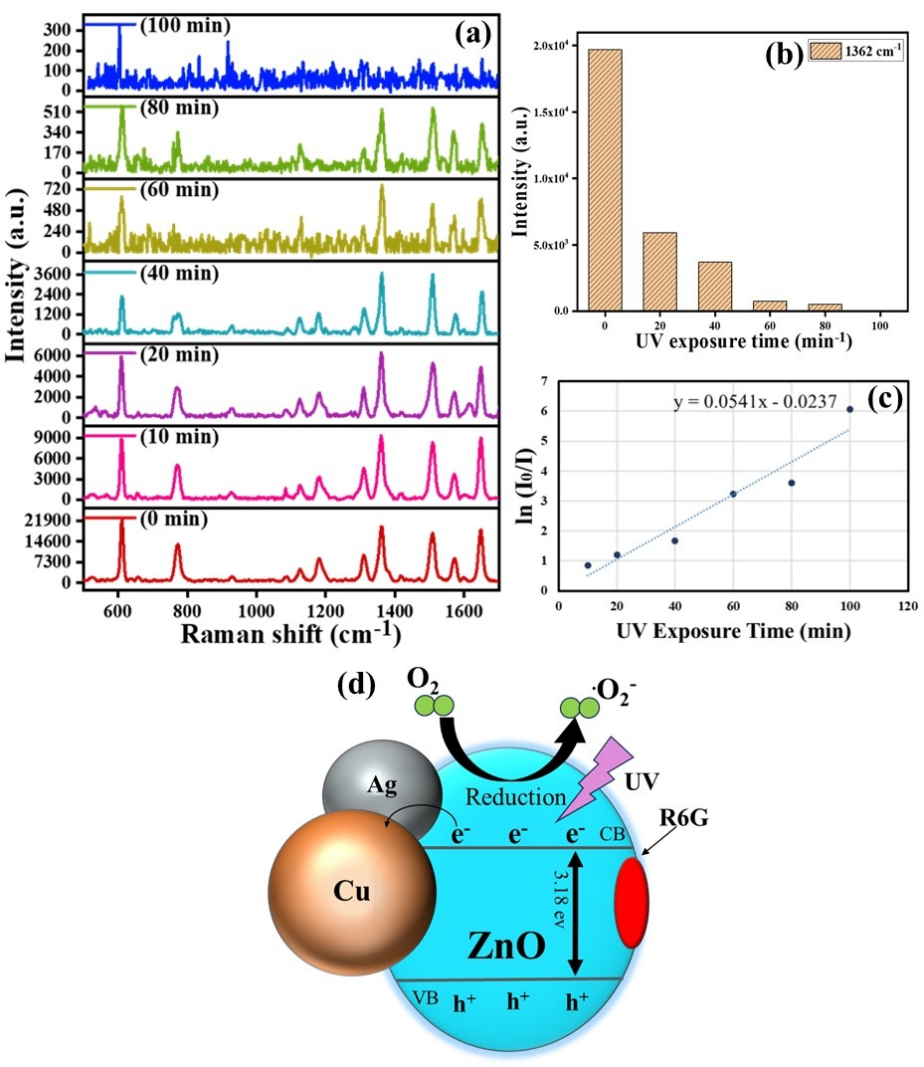
(a) SERS spectra of R6G with concentration of 10^−8^ M adsorbed on the ZAC 55 surface for different UV light exposure time span (0–100 mins) (photocatalytic degradation process); (b) the bar representation of R6G peak intensity at 1365 cm^−1^ with different UV exposure time; (c) Represents the calculation of first‐order degradation rate constant from the ln (Io/I) vs. UV exposure time, (d) represents the mechanism of photocatalytic degradation process.

Non‐destructive, self‐cleaning of a SERS substrate is one of the most important parameters to make it again reusable, which reduces the cost of the substrate. By exposing the substrate under radiation (365 nm), we investigated the reusability scope of the substrate, where photocatalytic behavior of ZnO resulted into the degradation of the adsorbed R6G molecule. Figure 9(a) displays the SERS spectra of 10^−^⁸ M R6G adsorbed onto ZAC55, recorded after each successive UV exposure for different period of time. A steady, gradual decrease in SERS intensity of R6G was observed with increasing UV exposure time, indicating the successful photocatalytic degradation of R6G molecules by ZAC55 composite. After 100 minutes of UV exposure, the Raman peaks disappeared completely (Figure 9b), signifying the complete degradation and removal of R6G. This photo‐assisted degradation process could be explained by the Langmuir‐Hinshelwood mechanism, following pseudo‐first‐order kinetics.^[51]^ Figure 9(c) shows the plot of ln(I₀/I) versus UV irradiation time, and from the slope the calculated value of the pseudo‐first‐order degradation rate constant (k) was found to be 0.0541 min^−1^.

ZnO is a well‐known photocatalyst for degrading organic molecules, and its photocatalytic efficiency can be significantly improved by forming a Schottky junction with metal nanoparticles.[^52^, ^53^] In this study, R6G dye was used as a model organic molecule adsorbed onto the substrate. Upon UV light exposure, the electrons were transferred from the conduction band (CB) of ZnO to the noble metals (Ag, Cu) through the Schottky barrier, leading to charge redistribution between ZnO and the metals,[^27^, ^54^] which enhanced photocatalytic activity. Photoexcited electrons from R6G molecules were transferred to the noble metals or the CB of ZnO, as well as to shallow trap levels within ZnO's band gap. Simultaneously, after photoexcitation, ZnO allowed electrons from the valence band (VB) to jump to the CB, leaving behind holes in the VB and generating electron‐hole pairs (excitons, e^−^‐h^+^). These e^−^‐h^+^ pairs are highly reactive species. The noble metals (Ag and Cu) acted as electron extraction sites, while the holes remained in ZnO, facilitating better charge separation. A visual representation of the photocatalytic mechanism of the ZnO−Ag−Cu system is depicted in Figure 9d. Due to the LSPR effect, increased efficiency of light absorption in noble metals and the charge separation in the ZnO synergistically improved the formation of reactive oxygen species (ROS), and may facilitated electron transfer processes. But the effect of LSPR was quite negligible because the SPR wavelength of this noble metals were quite higher than the UV excitation wavelength ~365 nm. The photo‐degenerated e^−^s present in the CB produced superoxide radicals (⋅O_2_^−^) by reacting with oxygen molecule present in the environment. The superoxide radicals are powerful ROS which plays significant role in the degradation of R6G into CO_2_ and other small molecules through oxidative reactions.[^55^, ^56^] The probable reactions involved in the decomposition of R6G, during the photocatalytic degradation process by ZnO−Ag−Cu composite are as follows:

- ZnO+hv (UV)→e^−^ (electron in CB)+h^+^ (hole in VB).
- O_2_+e^−^→ ⋅O_2_^−^
- R6G+⋅O_2_^−^→Degraded products/small molecules.

### 2.9 Reusability Study of SERS Substrate by UV‐Assisted Self‐Cleaning

**Figure 10:**
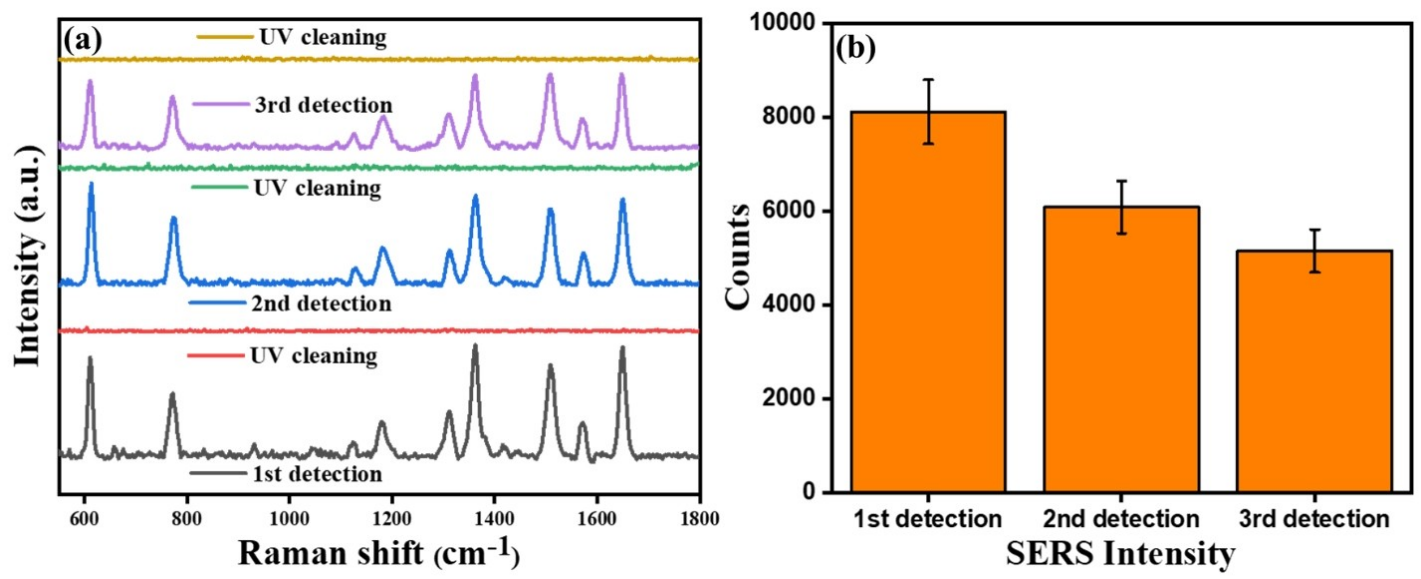
(a) Raman spectra of R6G (10^−8^ M) adsorbed on ZAC 55 substrate before and after the UV assisted self‐cleaning process for 3 cycles. Each cycle consists of adsorption of R6G molecules by drop casting method followed by UV irradiation for 100 min; (b) SERS intensity of 1653 cm^−1^ peak of R6G before and after UV cleaning, demonstrating reusability.

The rapid photodegradation of R6G molecules has prompted efforts to reuse the substrate for multiple cycles of SERS‐based molecular detection. Figure 10 (a) illustrates the SERS spectra of R6G molecules (10^−8^ M) before and after a UV‐assisted self‐cleaning process on the ZAC 55 substrate. In the first cycle, strong Raman signals were observed, while no Raman signals were detected after 100 minutes of UV exposure, indicating the complete decomposition of R6G molecules and effective self‐cleaning of the substrate. Upon re‐adsorption of a 10^−8^ M solution of R6G onto the cleaned substrate again, the Raman signals of R6G were restored, confirming the substrate's retention of SERS activity even after UV treatment (2^nd^ detection). The reusability study^[57]^ was successfully repeated for three cycles, showing consistent recovery of R6G signals, albeit with a slight decline in SERS intensity during subsequent detections compared to the first cycle. However, the heterogenous nature of the hotspots (Figure 6) also contributed to this deviation, as the SERS spectra could not be collected from the exact same spot after the UV treatment. These findings highlight the robustness and potential of the ZAC 55 substrate for repeatable SERS applications, offering a sustainable and cost‐effective approach for sensing molecular analytes. This demonstrates that the substrate can maintain its structural integrity and SERS efficiency, even after multiple cycles of UV cleaning, further enhancing its value for practical analytical applications.

## 3 Conclusions

Semiconductor‐bimetallic ZnO−Ag−Cu (ZAC) composite of various compositions were synthesized, characterized in detail and successfully used as SERS substrates. While, ZnO NRs were prepared by simple hydrothermal method, semiconductor oxide composites were successfully synthesized with variation in Ag and Cu content, via impregnation method using PVP as a stabilizing agent. Using the reporter molecule R6G under 532 nm excitation, the best SERS substrate was found to be ZAC55, which contained same amount of Ag and Cu in the composite. Whereas, any imbalance in the composition (ZAC37, ZAC64 and ZAC73) resulted in much lesser SERS performance. The XPS analysis of ZAC55 revealed that Ag remained in its active plasmonic state with least oxidation, while higher oxidation of Ag was recorded for ZAC73 and ZAC37. ZAC55 registered SERS EF of 6x10^6^ and the same substrate showed extraordinary SERS efficiency by detecting R6G at ultralow concentrations of 1 aM and MB at 1 fM. While, no SERS signal of R6G was detected using either bare ZnO NRs or ZC composite, but ZA, with the incorporation of Ag into the ZnO lattice, produced decent SERS performance with an LOD of 10^−12^ M for R6G. The presence of Cu in its oxidised form with ZnO, could not able to produce any SERS signal in ZC due to lack of LSPR efficiency. Thus, it can be rationalized that the incorporation of both Ag and Cu nanoparticles into the ZnO matrix improved the material's plasmonic, structural and surface properties, enhancing its overall SERS performance. Additionally, a comparison of photoluminescence intensity and lifetime of pure ZnO with the composites, indicated about a superior charge transfer process in ZAC 55. Use of such metal‐semiconductor composite helps in reducing the substrate cost due to the photocatalytic activity of ZnO, as we achieved complete photocatalytic degradation of the 10^−8^ M R6G adsorbed onto ZAC55 under UV exposure of 100 mins, implying the reuse of the single substrate for several times. Overall, the synergy between the metal additives and ZnO resulted in enhanced plasmonic efficiency, improved charge transfer and useful photocatalytic property, rendering semiconductor‐bimetallic heterojunction ZnO−Ag−Cu as a highly sensitive and self‐cleanable SERS substrate. Further research is going on in our laboratory to optimize the composite's synthesis parameters and use of other semiconductor oxide composite to further enhance SERS efficiency.

## Conflict of Interests

The authors declare no competing financial interest.

4

## Supporting information

As a service to our authors and readers, this journal provides supporting information supplied by the authors. Such materials are peer reviewed and may be re‐organized for online delivery, but are not copy‐edited or typeset. Technical support issues arising from supporting information (other than missing files) should be addressed to the authors.

Supporting Information

## Data Availability

The data that support the findings of this study are available in the supplementary material of this article.
