# Supplementary material for: Semiconductor‐Bimetallic Plasmonic Heterojunction ZnO−Ag−Cu as Reusable SERS Substrate with Attomolar Detection Limit
Source: Chem Asian J. 2025 Feb 18;20(9):e202401580. doi: 10.1002/asia.202401580 (PMC12067856; doi:10.1002/asia.202401580)
Supplement: Supplementary file 1 — Supporting Information [file ASIA-20-e202401580-s001.pdf]

Chemistry - An Asian Journal

Supporting Information

Semiconductor-Bimetallic Plasmonic Heterojunction ZnO–Ag–Cu as Reusable SERS Substrate with Attomolar Detection Limit

Rojalin Behera and Amit Nag*

Supporting Information

Semiconductor-bimetallic plasmonic heterojunction ZnO-Ag-Cu as reusable SERS substrate with attomolar detection limit

Rojalin Behera and Amit Nag*

Department of Chemistry, BITS Pilani, Hyderabad campus,500078, India

*Email: amitnag@hyderabad.bits-pilani.ac.in

Contents:

Figure S1: Image of the different composites in their powder form

Figure S2: XRD spectra of ZC and ZAC55 showing the presence of Cu peak in the ZAC55 composite

Figure S3: XPS spectra of composites

Figure S4: Extinction Spectra of all ZAC composites

Figure S5: Tauc plots of all the prepared sample (ZnO, ZA, ZAC 73, ZAC 55, ZAC 37, ZC).

Figure S6: Optical image for ZAC 55 substrate, collected under Raman microscope

Figure S7: No SERS signal from blank ZAC 55 substrate

Figure S8: SERS spectra of BTA adsorbed onto ZAC 55.

Calculation S1: SERS Enhancement Factor Calculation of ZAC 55

Figure S9: Reproducibility check of ZAC 55 substrate

Figure S10: Photoluminescence Decay curves of ZnO, ZA, ZAC 55

Table S1: Lifetime values of ZnO, ZA, ZAC 55

Figure S11: SERS-based R6G detection using ZA, ZAC 37 and ZAC 73

Figure S12: FE-SEM images and corresponding EDX spectra of ZAC 37 and ZAC 73

Table S2: Assignment of R6G vibrational peaks

Table S3: Assignment of MB vibrational peaks

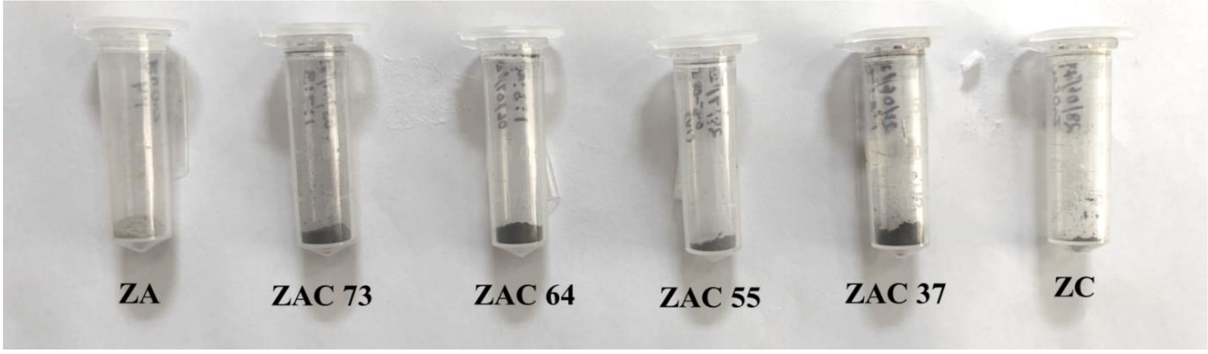

Figure S1: Image showing different composites in their powder form.

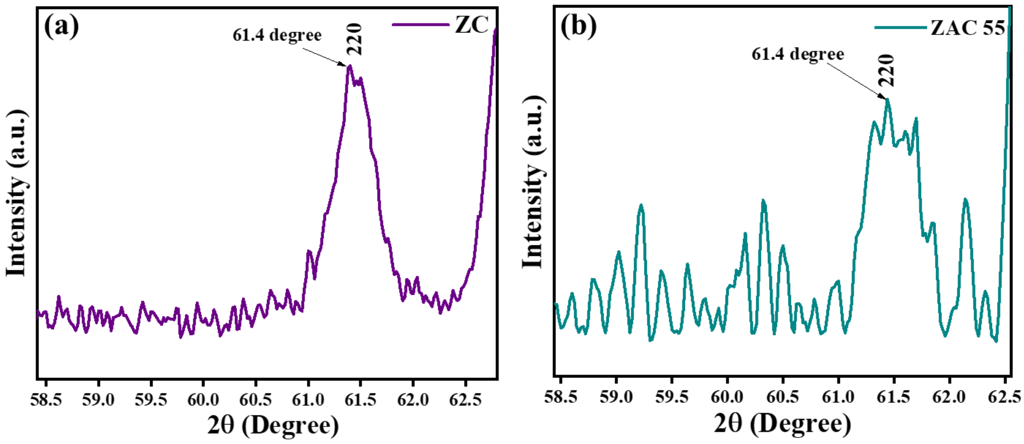

Figure S2: XRD spectra of (a) ZC, (b) ZAC55 showing the presence of Cu peak in the ZAC55 composite.

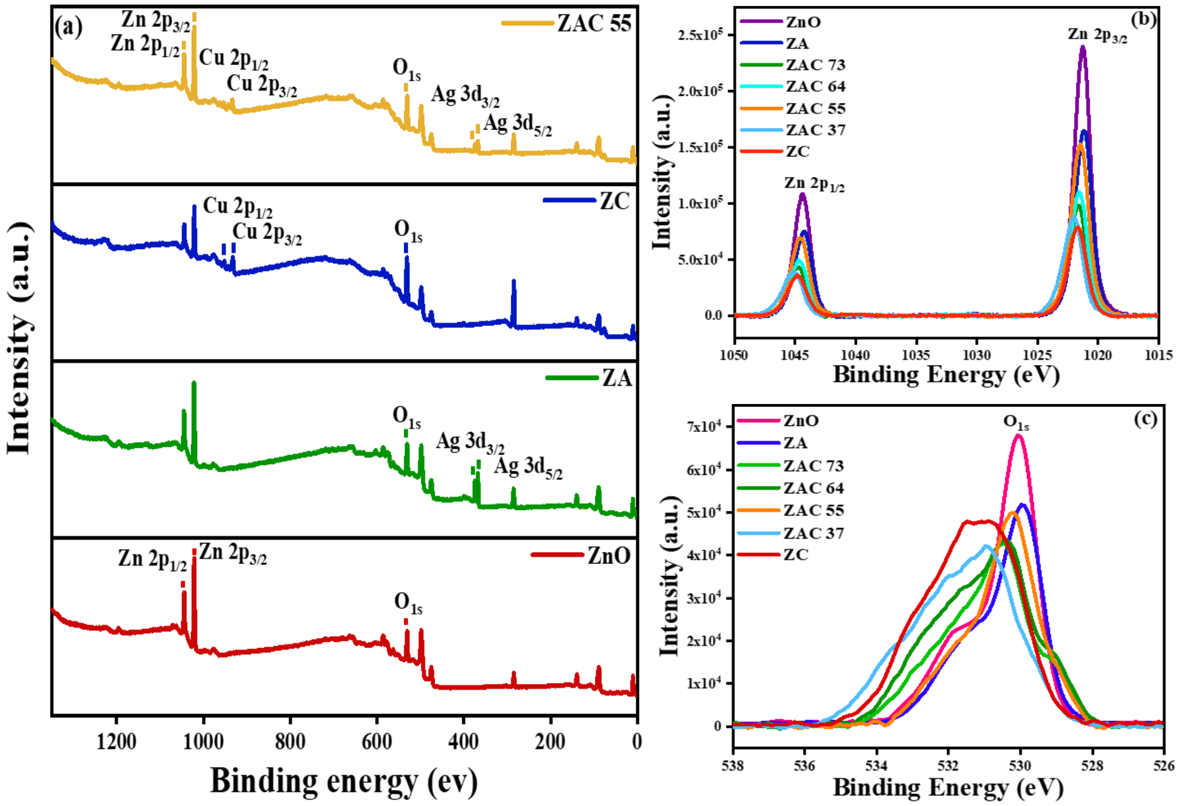

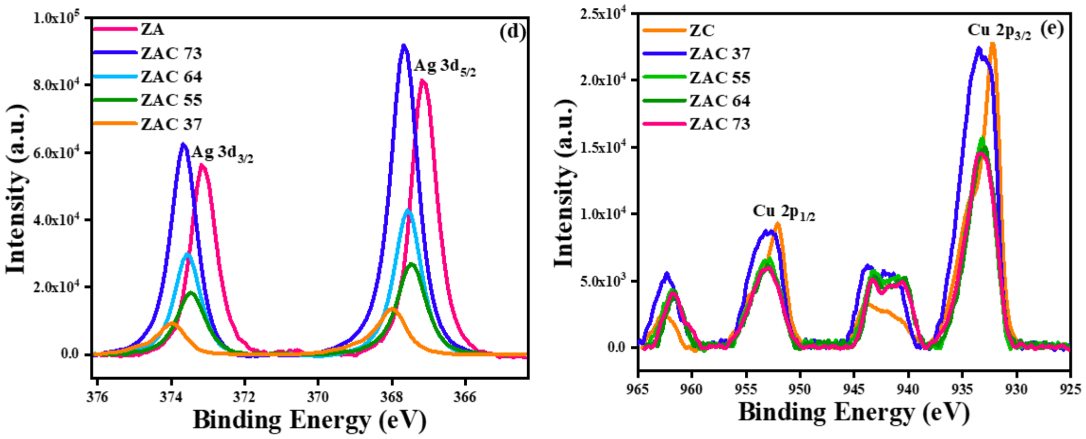

Figure S3: (a) represents the stacked XPS survey spectra of different composites; comparison of all the composites' XPS spectra including ZAC64: (b) Zn 2p (c) O 1s (d)Ag 3d (e) Cu 2p.

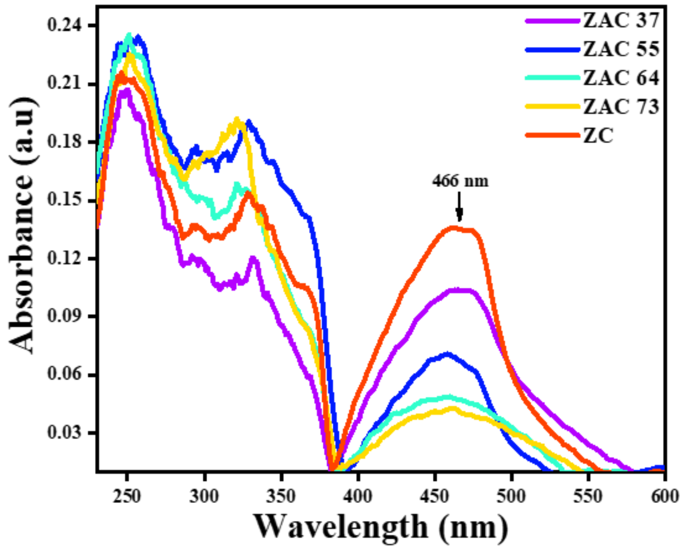

Figure S4: Extinction Spectra of all ZAC composites.

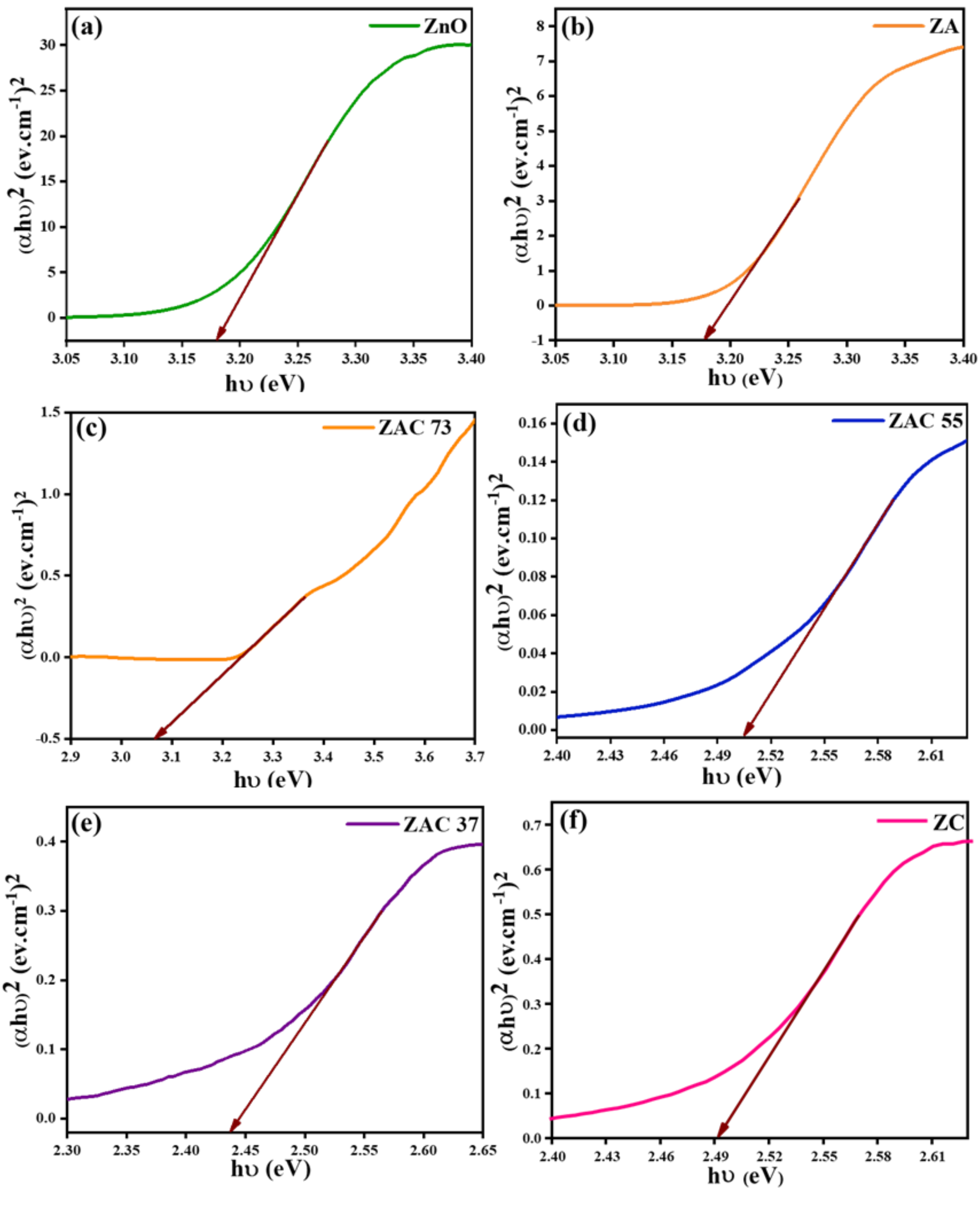

Figure S5: Tauc plots of (a) ZnO, (b) ZA, (c) ZAC 73, (d) ZAC 55, (e)ZAC 37 (f) ZC.

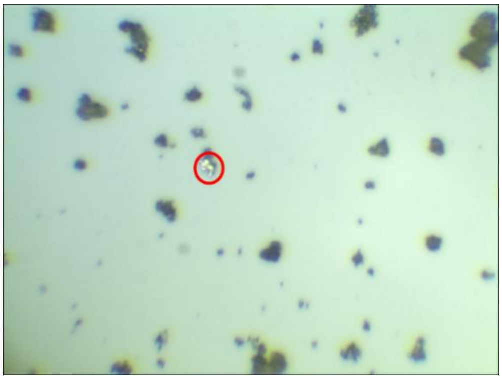

Figure S6: Optical image collected under Raman microscope, using 50X Objective for ZAC 55 substrate after drop cast, where the marked glowing portion results into good SERS signal.

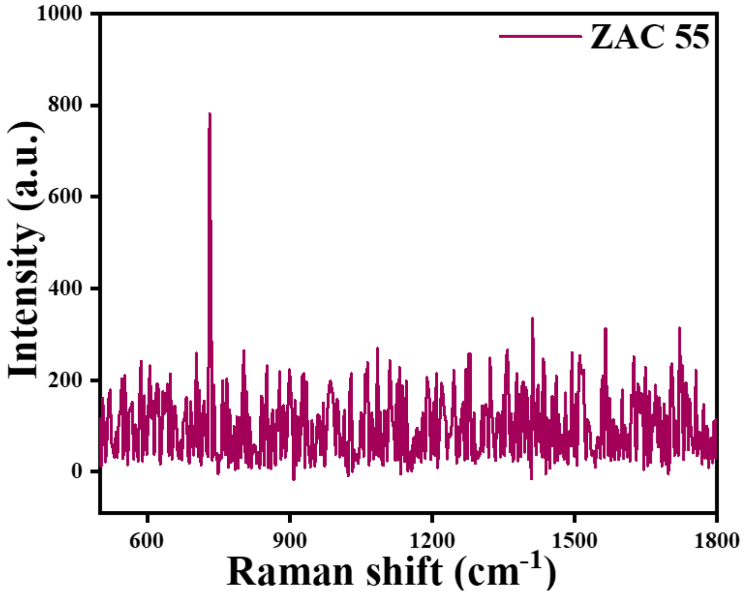

Figure S7: No SERS signal is obtained from blank ZAC 55, before adsorbing R6G onto the substrate's surface.

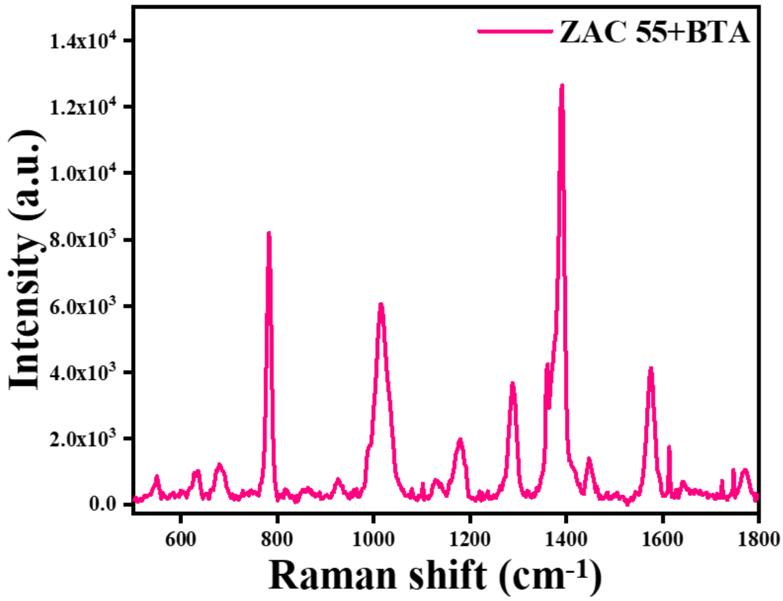

Figure S8: SERS spectra of BTA adsorbed onto the surface of ZAC 55 composite.

Calculation S1: SERS Enhancement factor (EF) calculation of ZAC55

The enhancement factor, was calculated as mentioned in the paper by the following formula:

EF= (I_{SERS} / IRaman) × (N_{bulk} / $N_{surface}$);

We have used the following experimental conditions to acquire the Raman spectra.

Laser beam spot size (diameter): 2 μm, radius (r) = 1 μm

Rayleigh range (h) = $\pi \omega_0{}^2/\lambda$ = 3.14 × 4 × 10 $^{-12}m^2$ /532 × 10 ^{-9}m = 23.6 μm

Laser spot area = A = 3.14 x 10^{-8} cm^2

Focal volume = A x h = 74.1 x 10^{-12} cc

$N_{surface}$ is the product of C, the surface density of BTA and A, the laser spot area respectively.

Surface density of BTA for $N_{surface}$ calculation:

40 μL of 10 μM BTA solution was spread over a ZAC 55 composite, having area of ~4 cm^2. This yielded the surface density of BTA on the SERS substrate is 6 x 10^{13} molecules/cm^2, assuming homogeneous spreading.

So, **$N_{surface}$ = 6 x 10^{13} x 3.14 x 10^{-8} = 18.8 x 10^5.**

Raman spectrum of solid BTA powder is used for N_{bulk} calculation. Considering, density of BTA as 1.36 g/mL and molecular weight 119.127 gm/ mol, number of BTA molecules present in the focal volume (N_{bulk}) is:

$N_{Bulk} = \boldsymbol{A h \rho / m} = \mathbf{74.1 \times 10^{-12} \times 1.36 \times 6.02 \times 10^{23} / 119.127 = 5.1 \times 10^{11}}$

so, $N_{Bulk}/N_{Surface} = \mathbf{2.7 \times 10^5}$

I_{SERS}/ I_{Raman} is evaluated to be considering the integrated intensity of BTA, for 2 different bands (~ 782 cm^{-1} and 1387 cm^{-1}) from surface enhanced and normal Raman spectrum as following. **The enhancement factor of ZAC 55 is ~ 3.9 × 10^6 and 6.2 × 10^6 using, ~ 782**

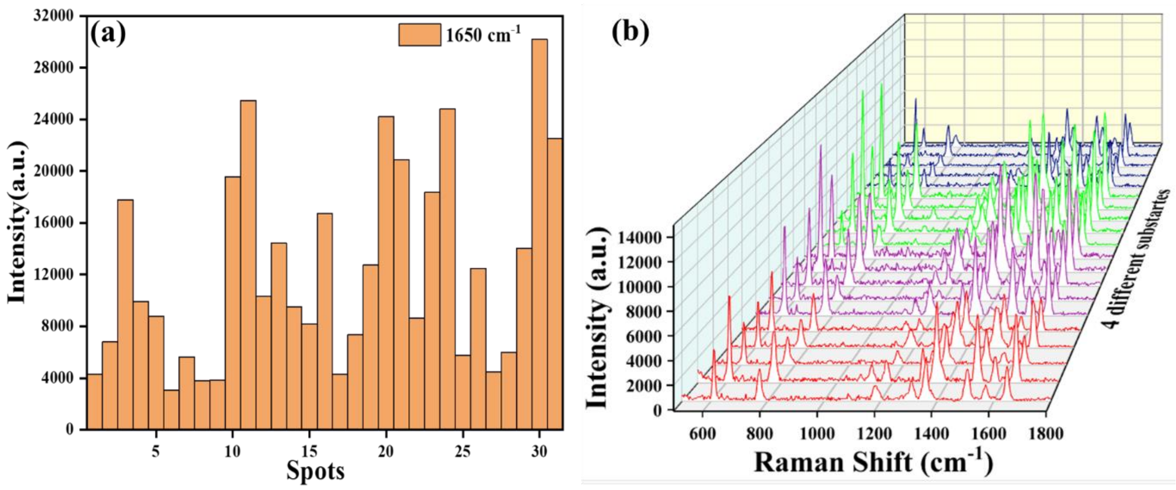

cm^{-1} and 1387 cm^{-1} band, respectively.

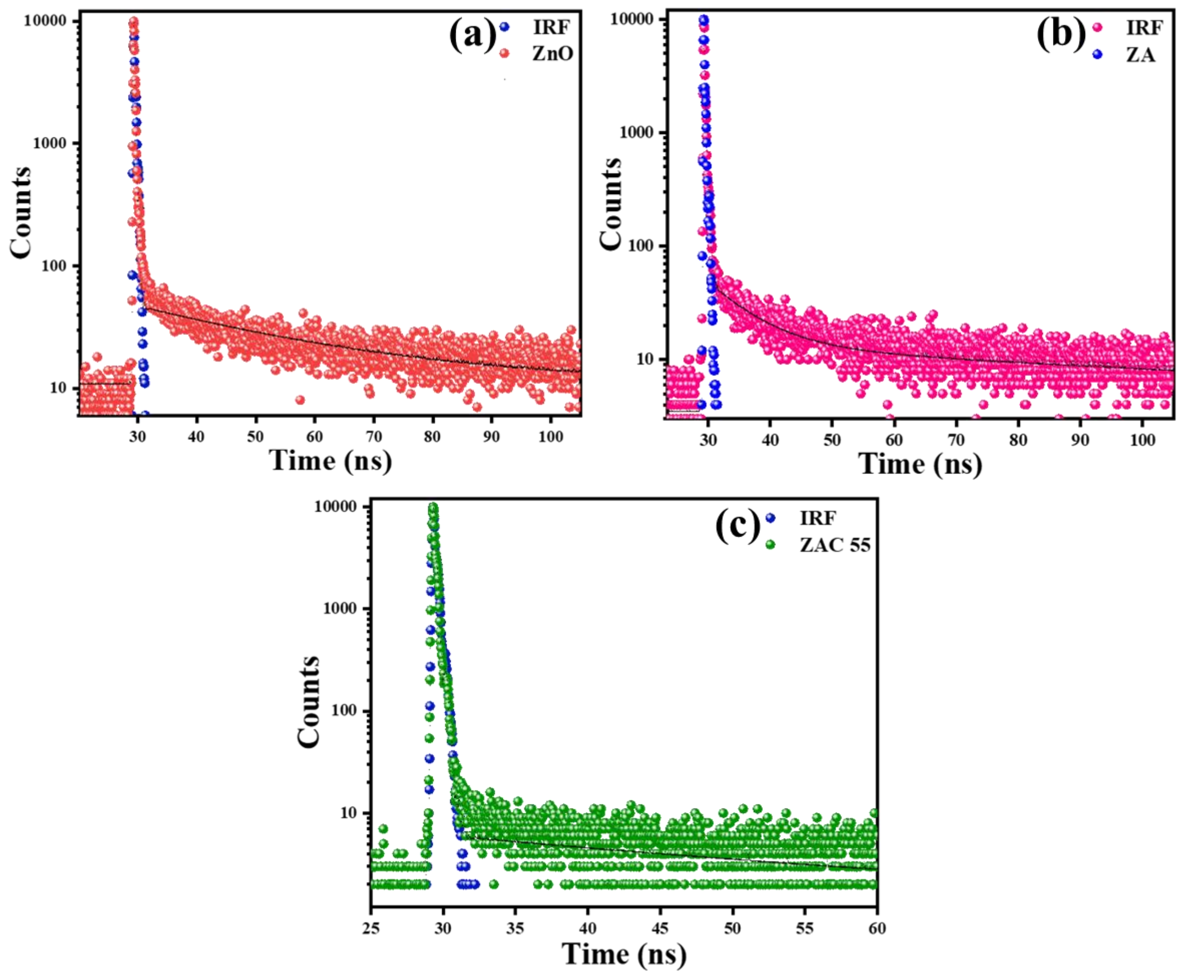

Figure S9: (a) Bar plot of the SERS intensity of 31 random detection points on the ZAC 55 substrate after adsorbing 10^{-8}M of R6G onto it, (b) SERS intensity of 5 detection point on the 4 different ZAC 55 drop casted substrate after adsorbing 10^{-8}M of R6G onto it.

Figure S10: Fitted photoluminescence decay curves of (a) ZnO, (b) ZA, (c) ZAC 55.

Table S1: Lifetime values of ZnO, ZA, ZAC 55

**Sample name**	**λ_{ex} (nm)**	**λ_{em} (nm)**	**τ_1 (ns)**	**τ_2 (ns)**	**τ_3 (ns)**	**$\propto_1$**	**$\propto_2$**	**$\propto_3$**	**χ^2**	**Average lifetime(ns)**
ZnO	377	560	28.650	0.120	0.120	0.001	0.499	0.500	1.66	0.12
ZA	377	560	6.798	93.45	0.02	0.02	0.08	0.9	1.24	0.08
ZAC 55	377	560	27.52	0.13	0.06	0.02	0.25	0.73	1.21	0.07

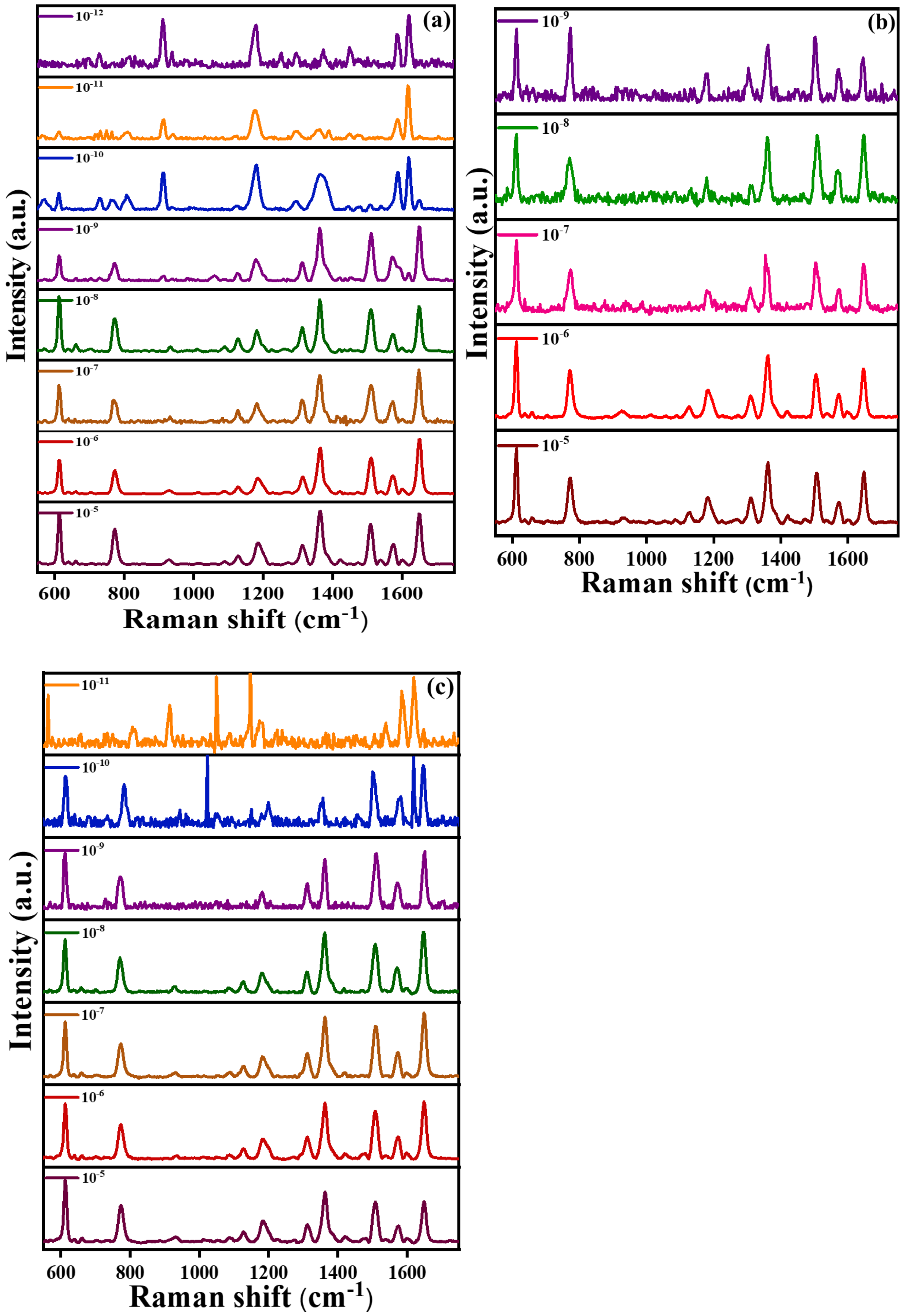

Figure S11: SERS-based R6G detection using (a) ZA (b) ZAC 37(c) ZAC 73.

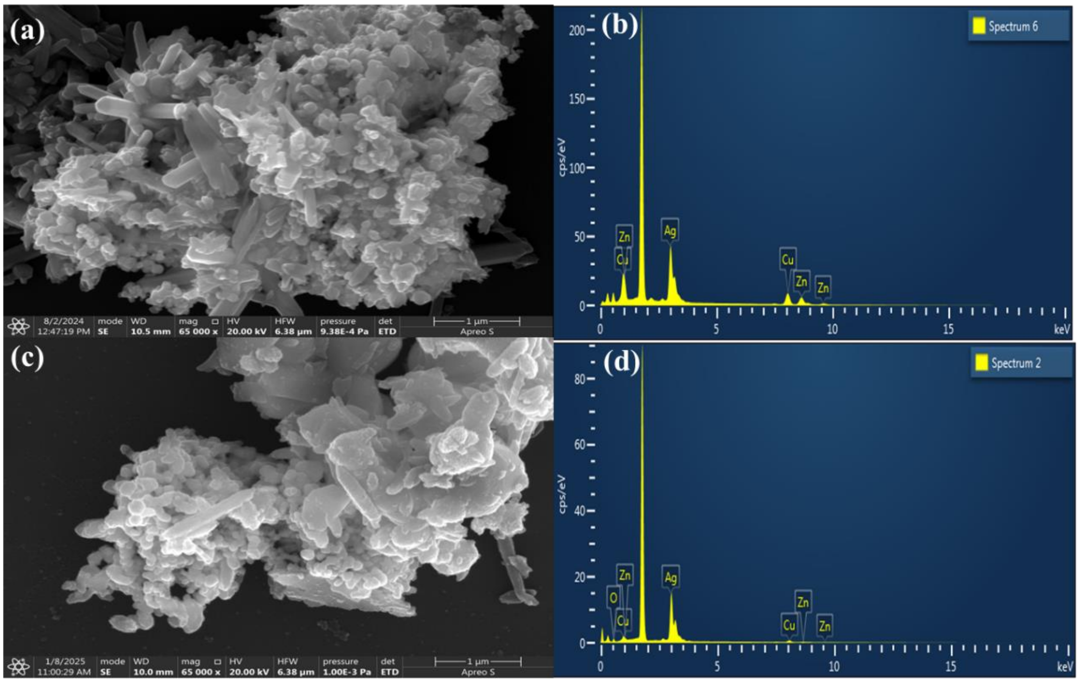

Figure S12: FE-SEM images and corresponding EDX spectra of ZAC 37 (a, b) and ZAC 73 (c, d).

Table S2: Assignment of R6G vibrational peaks, *s, m, w, v, sh represents to strong, medium, weak, very, shoulder, respectively.

Raman shift (cm^{-1})	FTIR (cm^{-1})	Functional groups
613 cm^{-1} (s)	-	C-C-C ring in plane bending
-	724 cm^{-1} (w)	
	755 cm^{-1} (w)	
777 cm^{-1} (sh)	-	C-H out plane bending
770 cm^{-1} (vw)		C-H out plane bending
809 cm^{-1} (vw)	801 cm^{-1} (w)	External Group Mode (EGM)
923 cm^{-1} (w)	910 cm^{-1} (s)	C-H out plane bending
936 cm^{-1}(vw)	931 cm^{-1}(w)	C-H out plane bending
1127 cm^{-1} (w)	1119 cm^{-1} (w)	
1184 cm^{-1} (m)	1175 cm^{-1} (s)	C-H in plane bending
1279 cm^{-1} (sh)		C-O-C stretching
	1292 cm^{-1} (w)	C-H deformation (EGM)
1314 cm^{-1} (m)		C-H bond stretching
1367 cm^{-1}(s)	1367 cm^{-1} (s)	C-C aromatic stretching
1432 (vw)	1436 cm^{-1} (w)	EGM ethylamino group
1510 cm^{-1} (s)		aromatic C-C stretching mode
1540 cm^{-1} (vw)	1536 cm^{-1} (w)	EGM
1575 cm^{-1} (m)	1586 cm^{-1} (s)	aromatic C-C stretching mode
1607 cm^{-1} (sh)	1618 cm^{-1} (s)	EGM
1653 cm^{-1} (s)		aromatic C-C stretching mode

Table S3: Assignment of MB vibrational peaks.

**Raman shift (cm^{-1})**	**Functional groups**
1397 cm^{-1}	C-H in plane ring deformation
1624 cm^{-1}	C-C stretching
